# Naïve-like pluripotency to pave the way for saving the northern white rhinoceros from extinction

**DOI:** 10.1038/s41598-022-07059-w

**Published:** 2022-03-08

**Authors:** Vera Zywitza, Ejona Rusha, Dmitry Shaposhnikov, Jorge Ruiz-Orera, Narasimha Telugu, Valentyna Rishko, Masafumi Hayashi, Geert Michel, Lars Wittler, Jan Stejskal, Susanne Holtze, Frank Göritz, Robert Hermes, Jichang Wang, Zsuzsanna Izsvák, Silvia Colleoni, Giovanna Lazzari, Cesare Galli, Thomas B. Hildebrandt, Katsuhiko Hayashi, Sebastian Diecke, Micha Drukker

**Affiliations:** 1 Technology Platform Pluripotent Stem Cells, Max‐Delbrück‐Center for Molecular Medicine in the Helmholtz Association (MDC), 13125 Berlin, Germany; 2 Present Address: Induced Pluripotent Stem Cell Core Facility, Helmholtz Zentrum München, 85764 Neuherberg, Germany; 3 Cardiovascular and Metabolic Sciences, Max-Delbrück-Center for Molecular Medicine in the Helmholtz Association (MDC), 13125 Berlin, Germany; 4 Department of Stem Cell Biology and Medicine, Graduate School of Medical Sciences, Kyushu University, Maidashi 3-1-1, Higashi-ku, Fukuoka, 812-8582 Japan; 5 FEMTransgenic Technologies, Charité, 13125 Berlin, Germany; 6 Department of Developmental Genetics, Max Planck Institute for Molecular Genetics, 14195 Berlin, Germany; 7 ZOO Dvůr Králové, Štefánikova 1029, 544 01 Dvůr Králové nad Labem, Czech Republic; 8 Leibniz Institute for Zoo and Wildlife Research, 10315 Berlin, Germany; 9 Present Address: Mobile DNA, Max-Delbrück-Center for Molecular Medicine in the Helmholtz Association (MDC), 13125 Berlin, Germany; 10 Laboratory of Reproductive Technologies, Avantea, 26100 Cremona, Italy; 11 Fondazione Avantea, 26100 Cremona, Italy; 12 Faculty of Veterinary Medicine, Freie Universität Berlin, 14163 Berlin, Germany; 13 Division of Drug Discovery and Safety, Leiden Academic Centre for Drug Research (LACDR), Leiden University, 2300 RA Leiden, The Netherlands

**Keywords:** Biological techniques, Cell biology, Developmental biology, Stem cells

## Abstract

The northern white rhinoceros (NWR) is probably the earth’s most endangered mammal. To rescue the functionally extinct species, we aim to employ induced pluripotent stem cells (iPSCs) to generate gametes and subsequently embryos in vitro. To elucidate the regulation of pluripotency and differentiation of NWR PSCs, we generated iPSCs from a deceased NWR female using episomal reprogramming, and observed surprising similarities to human PSCs. NWR iPSCs exhibit a broad differentiation potency into the three germ layers and trophoblast, and acquire a naïve-like state of pluripotency, which is pivotal to differentiate PSCs into primordial germ cells (PGCs). Naïve culturing conditions induced a similar expression profile of pluripotency related genes in NWR iPSCs and human ESCs. Furthermore, naïve-like NWR iPSCs displayed increased expression of naïve and PGC marker genes, and a higher integration propensity into developing mouse embryos. As the conversion process was aided by ectopic *BCL2* expression, and we observed integration of reprogramming factors, the NWR iPSCs presented here are unsuitable for gamete production. However, the gained insights into the developmental potential of both primed and naïve-like NWR iPSCs are fundamental for in future PGC-specification in order to rescue the species from extinction using cryopreserved somatic cells.

## Introduction

The world is facing a sixth mass extinction, which is directly and indirectly driven by human activities^1–3^. For critically endangered mammals with small population sizes and low genetic diversity, conventional conservation strategies such as habitat protection and captive breeding may not be enough to restore a genetically healthy and self-sustainable population. At the same time, the seminal increase in understanding developmental biology in combination with technological advancements in stem cell biology create innovative opportunities to circumvent the inevitable extinction of diverse species of large mammals. In this respect, female somatic cells could in principle be used as a source for induced pluripotent stem cells (iPSCs), which in turn could be differentiated into oocytes in vitro, fertilized and transferred to surrogates of same or related species^4,5^. A crucial first step for accomplishing this goal is to gain knowledge on developmental regulation and differentiation mechanisms of PSCs derived from critically endangered species, which fulfill certain prerequisites indicating successful implementation of the proposed modality. *Rhinocerotidae spp.* are good candidates because (1) this family includes highly endangered as well as less threatened species, which could serve as surrogates, (2) important advancements have been made in assisted reproductive technology (ART)^6–9^, and (3) iPSCs have already been derived from specimen of the functionally extinct northern white rhinoceros (NWR, *Ceratotherium simum cottoni*) using standard techniques for reprogramming and maintaining human iPSCs^10,11^.

In vitro gametogenesis, the generation of gametes in cell culture from PSCs, has been adapted to several mammalian species (reviewed in Ref.^12^). However, the capacity and efficiency to produce functional gametes in vitro varies between species necessitating protocol adaption for each species individually. As a first step, primordial germ cells (PGCs) have to be specified from PSCs. The state of pluripotency, in which PSCs reside, has been shown to be crucial for PGC competence (reviewed in Refs.^12,13^). In vitro, features of naïve (also called ground) and primed state pluripotency are interchangeable through the application (or withdrawal) of small inhibitor molecules and growth factors. Embryonic stem cells (ESCs) from both mouse and human are derived from the inner cell mass of the pre-implantation epiblast. However, while mouse ESCs resemble the naïve state of their in vivo counterpart, human ESCs and iPSCs strongly resemble mouse epiblast stem cells (EpiSCs), which are derived from the post-implantation epiblast and reside in primed state pluripotency. Through inhibition of both GSK3 and MEK signaling pathways in addition to LIF (2iL conditions), mouse ESCs can be maintained serum-free and stably in naïve state pluripotency. Along this line, human ESCs and iPSCs can be converted to naïve-like states by several combinations of inhibitors and growth factors (reviewed in Ref.^14^). Notably, typical primed state PSCs have little PGC competence, while cells at the transition from naïve to primed state readily generate PGCs^15,16^.

Here, we show the generation of NWR iPSCs by episomal plasmids^17^, and characterize their multilineage differentiation potential into cells of the three germ layers and trophoblast, which indicates high similarity to human primed state PSCs^18,19^. We demonstrate that NWR iPSCs acquire naïve-like pluripotency. The conversion process to naïve-like pluripotency was aided by ectopic overexpression of the anti-apoptotic gene *BCL2.* Furthermore, we show that NWR iPSCs contribute to tissues of the early developing mouse embryo, and that the integration propensity of NWR iPSCs into mouse blastocysts was increased in naïve culturing conditions. The classification of naïve-like NWR iPSCs is further supported by global transcriptomics, which revealed a similar expression profile of pluripotency related genes in naïve-like NWR iPSCs and naïve human ESCs. Taken together, these results create essential grounds for utilizing NWR iPSCs in an attempt to reverse the process of extinction.

## Results

### Virus-free generation of iPSCs from the NWR Nabire

**Figure 1:**
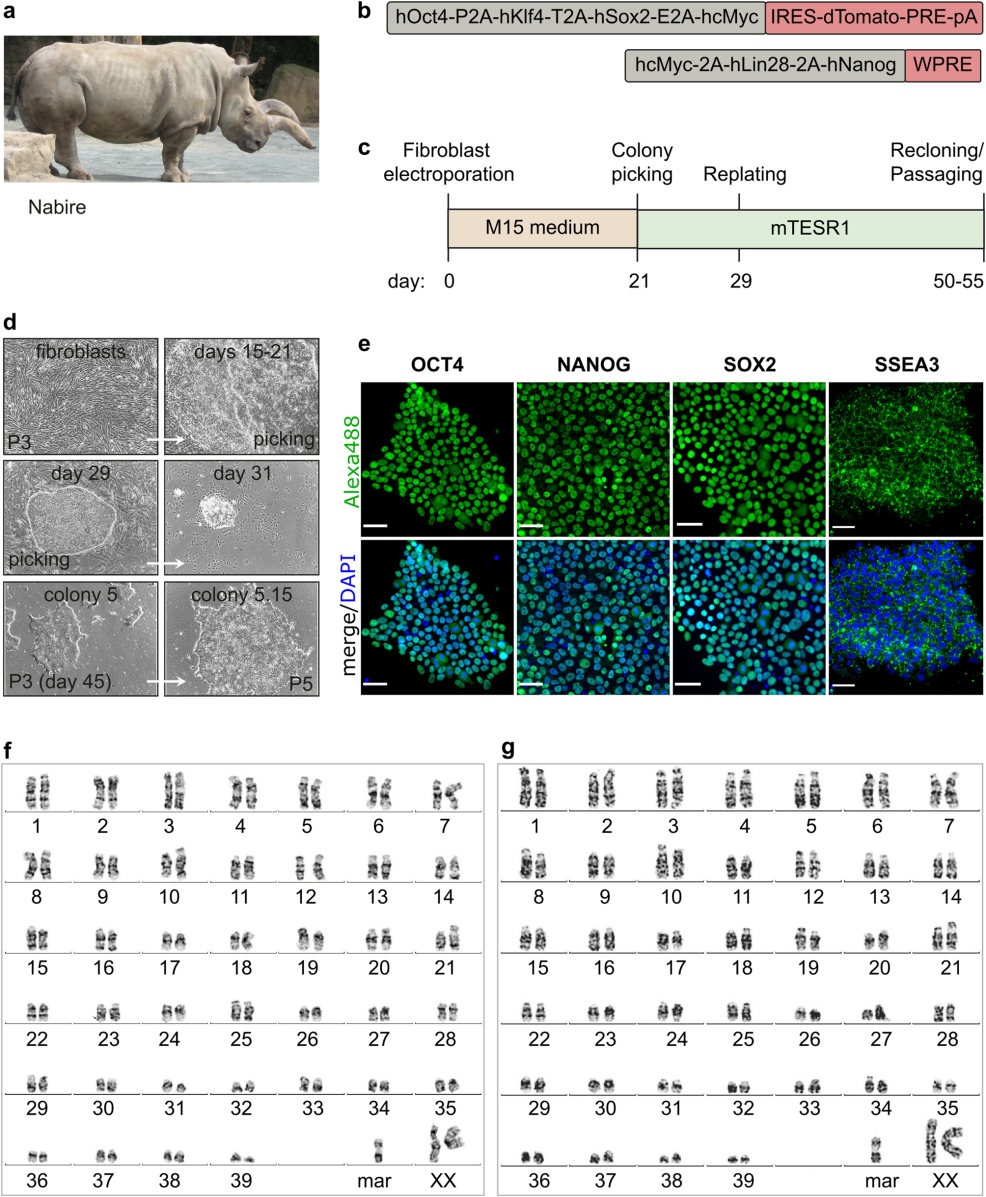
Generation and characterization of NWR iPSCs. See also Supplementary Figs. S1 and S2. (**a**) A photograph of the NWR Nabire. Author: Jan Robovský, Source: Groves et al.^23^; Permission: Copyright: © 2010 Groves et al. This is an open-access article distributed under the terms of the Creative Commons Attribution License, which permits unrestricted use, distribution, and reproduction in any medium, provided the original author and source are credited. Changes: here, only Nabire is shown. (**b**) Scheme of the reprogramming vectors coMIP247 (top) and pCXLE-hMLN (bottom). (**c**) Timeline of the reprogramming strategy of NWR skin fibroblasts to iPSCs. (**d**) Representative cell images during reprogramming. All images were acquired with 10x magnification. Additional information in Supplementary Fig. S1. (**e**) Representative images of NWR iPSCs stained for the pluripotency markers OCT4, NANOG, SOX2, SSEA3. Scale bars: 50 µm. **(f,g) **Representative 2n = 81 chromosome complements of NWR Nabire skin fibroblasts (**f**) and NWR iPSCs (**g**). *mar* marker chromosome.

We generated iPSC lines from Nabire (Fig. 1a), a female NWR who died in 2015 at the age of 31 years in the ZOO Dvůr Králové in the Czech Republic. We used a mini-intronic plasmid (MIP) protocol based on two vectors, namely coMIP247 and pCXLE-hMLN (Fig. 1b), which support reprogramming of diverse cell types including adult human blood cells^17,20^. The NWR skin sample was processed similar to human skin biopsies (see “Methods” section), including cryopreservation of primary fibroblasts at passages 1 to 3. To induce reprogramming, primary NWR fibroblasts were electroporated with both vectors and cultivated in M15 medium containing LIF^21^ (Fig. 1c). The characteristic mesenchymal to epithelial transition (MET) took place within 7 days, and colonies became apparent around day 15 (Fig. 1d). Media was changed to three different pluripotency supporting conditions (mTeSR1, 2i-hLIF, and bFGF) and on day 21 individual colonies were transferred onto feeder-free Matrigel coated dishes. Out of the tested media, only mTeSR1 supported further expansion and establishment of iPSC lines (Supplementary Fig. S1). Approximately 15% of the mTeSR1 colonies exhibited robust growth and undifferentiated cell morphology, which strikingly resembled primed human PSCs (Fig. 1d). Three independent NWR iPSC colonies were further propagated feeder-free in mTeSR1 medium, and cryopreserved. Subsequent experiments were mainly performed with two NWR iPSC lines. They expressed the canonical pluripotency factors OCT4, NANOG, SOX2 and the pluripotency surface marker SSEA3 (Fig. 1e). Karyotyping by g-banding of NWR iPSCs and source fibroblasts revealed karyograms with 2n = 81 chromosomes comprising 79 autosomes (including one marker chromosome) and two X chromosomes (Fig. 1f,g). This is in line with previously published karyograms of Nabire (Stud# 789), her sister Najin (Stud# 943), and their wild-born father Sudan (Stud# 372)^22^.

To test if either of the two used reprogramming vectors integrated into the genome of the generated iPSC lines, we performed PCR and RT-PCR of gDNA and RNA extracted from NWR iPSCs. Primers were designed over the linker regions in the plasmids to prevent amplification of endogenous sequences. Gel-electrophoresis revealed integration and expression of coMIP247, but not pCXLE-hMLN (Supplementary Fig. S2a). Next, we performed southern blotting of digested genomic DNA isolated from NWR fibroblasts and iPSCs at different passages with a probe recognizing part of the dTomato sequence encoded by the coMIP247 plasmid. We observed a single band in NWR iPSCs, but not fibroblasts, indicating that coMIP247 integrated at a single side in the NWR genome (Supplementary Figs. S2b, S3). RNA-sequencing confirmed low expression of coMIP247 encoded genes (reads mapping to coMIP247 are approximately 1/3 and 1/10 of all expressed NWR and endogenous NWR genes associated with stemness, respectively, Supplementary Fig. S2c. On single gene level, the coMIP247 encoded reprogramming factors *hOCT4*, *hKLF4, hSOX2*, and *hcMYC* are expressed approximately 1/29, 3/1, 1/3 and 1/48 of their endogenous NWR counterparts, respectively (Supplementary Fig. S2d). On protein level, coMIP247 encoded dTOMATO was not detected in living NWR iPSCs and immunostaining revealed just a weak signal at the detection limit (Supplementary Fig. S2e,f).

### NWR iPSCs differentiate into cells of the three germ layers and trophoblast progenitors

**Figure 2:**
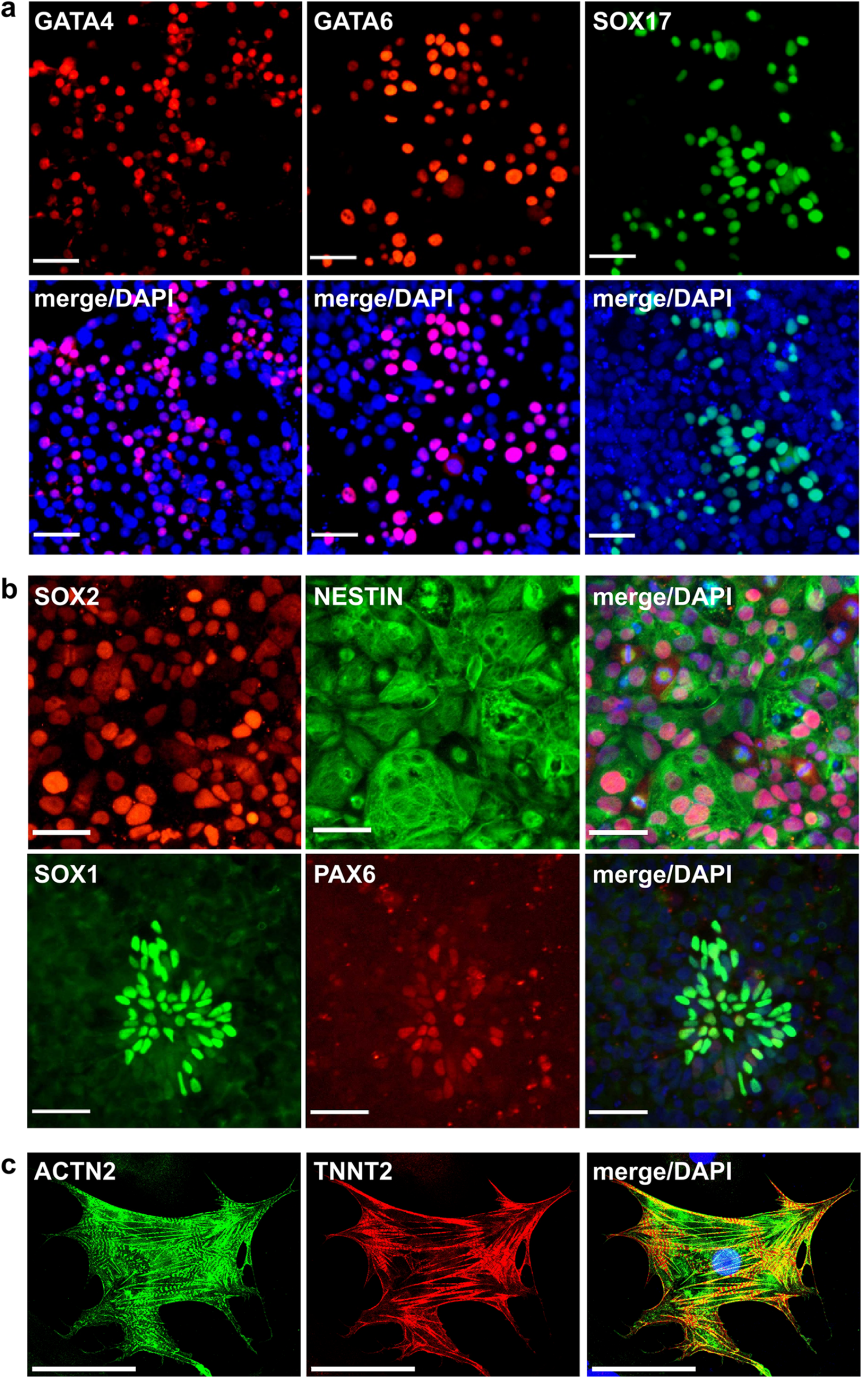
Three germ layer differentiation potential of NWR iPSCs. See also Supplementary Fig. S3, Supplementary Movie M1. Representative immunostainings for markers of the three germ layers upon differentiation of NWR iPSCs towards endoderm (**a**), neural precursors (ectoderm, (**b**)), and cardiomyocytes (mesoderm, (**c**)). Scale bars: 50 µm.

To assess pluripotency characteristics of NWR iPSCs, we tested whether the cells can differentiate into the three germ layers using protocols optimized for directed differentiation of human PSCs. Definitive endoderm progenitors were efficiently generated after 5 days of differentiation, and their identity was confirmed by immunostaining of GATA4, GATA6 and SOX17 (Fig. 2a). Towards ectoderm, NWR iPSCs were differentiated into neural progenitors using the dual SMAD inhibition protocol. We observed the formation of neural rosettes expressing SOX2, NESTIN, SOX1, and PAX6 after 7 days (Fig. 2b). Additionally, we differentiated NWR iPSCs into forebrain-like neurons expressing MAP2 by ectopic overexpression of NGN2 (Supplementary Fig. S4a). Mesodermal potency was demonstrated by generating beating cardiomyocytes via temporal modulation of WNT signaling (Supplementary Movie M1). The differentiation of cardiomyocytes was further confirmed by immunostaining of ACTN1 and TNNT2 (Fig. 2c). To test the extra embryonic potential of NWR iPSCs, we applied a protocol that promotes trophoblast differentiation through BMP4 exposure in human but not mouse ESCs^19^. Indeed, treatment with BMP4 for 72 h induced the expression of transcription factors regulating trophoblast specification in human ESCs, namely GATA2, GATA3, AP2-α and AP2-γ, as well as CDX2^24^ (Supplementary Fig. S4b) indicating a higher similarity of NWR iPSCs to human than mouse PSCs.

### NWR iPSCs adopt naïve-like pluripotency

**Figure 3:**
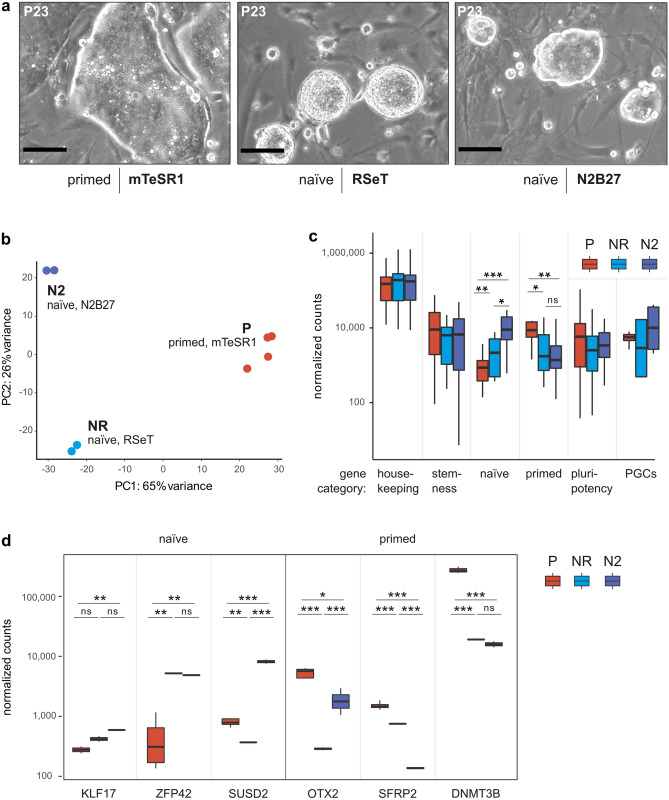
NWR iPSCs adopt naïve-like pluripotency. See also Supplementary Fig. S5 and Supplementary Table S1. (**a**) Representative images showing the cell morphology of NWR iBCL2-GFP-iPSCs grown on mouse embryonic fibroblasts in primed mTeSR1 (left)**,** and naïve conditions: RSeT (middle) and N2B27 (right) medium. Scale bars: 200 µm. (**b**) Principal component analysis (PCA) of transcriptome data from NWR iPSCs and NWR iBCL2-GFP-iPSCs cultured in primed and naïve conditions. (**c**) Comparative gene expression analysis of NWR iPSCs and NWR iBCL2-GFP-iPSCs cultured in primed and naïve conditions. Selected genes were grouped into categories and the average normalized expression counts were compared across samples. *, **, ***P value < 0.05, 0.01, 0.001, respectively (Wilcoxon test). Marker genes are provided in Supplementary Table S1. (**d**) Expression of marker genes in NWR iPSCs and NWR iBCL2-GFP-iPSCs cultured in primed and naïve conditions. *, **, ***P value < 0.05, 0.01, 0.001, respectively (adjusted P values DEseq2). *ns* not significant, *NR, N2* NWR iBCL2-GFP-iPSCs, naïve conditions, RSeT and N2B27 protocols, respectively, *P* NWR iPSCs primed conditions (mTeSR1).

Mouse and human PSCs can be cultivated in conditions promoting stable maintenance of primed and naïve pluripotency. Through activation and repression of signaling pathways, primed state cells can be converted to naïve state and vice versa (reviewed in Ref.^25^). Based on the morphological appearance of NWR iPSCs cultured in mTeSR1 medium (Fig. 1d), and the multi-lineage differentiation potential including trophoblast (Fig. 2, Supplementary Fig. S4), we hypothesized that they resemble the primed state of human iPSCs more than the classical naïve state of mouse ESCs. To investigate the feasibility of converting NWR iPSCs to naïve state, we tested two protocols: the commercial medium RSeT (based on Ref.^26^) and a N2B27 based protocol (modified from Ref.^27^). In both media conditions (and in both analyzed NWR iPSC lines), we observed spontaneous differentiation and cell death, which affects also conversion of human PSCs^28^. To improve cell survival and efficiency of the conversion process, we stably introduced the anti-apoptotic gene *BCL2* under control of an inducible promotor into one NWR iPSC line using a PiggyBac vector (resulting in NWR iBCL2-GFP-iPSCs) and supplemented the naïve culturing media with doxycycline to induce BCL2-GFP expression. This approach has been shown to improve the recovery of human and mouse ESCs from single cell dissociation and sorting^29^. Indeed, ectopic *BCL2* expression improved the conversion process to naïve-like pluripotency dramatically, and NWR iBCL2-GFP-iPSC colonies exhibited dome-shape morphology with shiny edges, which is characteristic for the naïve state, within 7 days of culturing in both naïve culturing conditions (Fig. 3a, Supplementary Fig. S5). In the presence of doxycycline, NWR iBCL2-GFP-iPSCs were stably maintained in a naïve-like state for more than 30 passages.

### Transcriptome analysis separates primed from naïve-like NWR iPSCs

To compare the gene expression profiles of NWR iPSCs in primed vs. naïve conditions, we performed total RNA-sequencing. Since the current NWR genome has not been properly assembled and annotated, we used the genome draft with partial annotation of the closely related southern white rhinoceros (SWR, *Ceratotherium simum simum*) as reference. The genetic divergence of NWR to SWR is estimated at 0.1%^30^. On average, about 85% of the NWR iPSCs RNA-sequencing reads mapped to the SWR genome. To quantify gene expression of NWR iPSCs, we first improved the existing SWR gene annotation by retrieving all protein-coding and non-coding RefSeq human, mouse, and horse genes from the University of California, Santa Cruz (UCSC) Genome Browser Database, and identifying their homologs in the SWR. Thereby, the final number of detected genes in the NWR transcriptome was increased by approximately 12,000 genes (in total 28,789 genes detected, see “Methods” section for details).

Next, we applied principal component analysis (PCA) to analyze the variability of NWR iPSCs in the three culturing conditions (Fig. 3b). PC1 explained 65% of the variance and divided naïve-like from primed samples. To analyze the expression of genes that are known to be associated with naïve and primed state, primordial germ cells (PGCs) as well as stemness and pluripotency in general, we averaged the expression levels of key marker genes (Supplementary Table T1) per condition (Fig. 3c). We found comparable expression levels of genes associated with housekeeping, stemness and pluripotency, respectively, across conditions, while the panel of primed stage genes was significantly lower expressed in cells cultured in naïve media. Vice versa, the panel of naïve marker genes was significantly higher expressed in naïve conditions. Additionally, we evaluated the expression of selected maker genes individually (Fig. 3d) and validated the results by RT-qPCR (Supplementary Fig. S6a, 15/18 trends agree). As expected, the primed marker genes *OTX2, SFRP2* and *DNMT3B* were significantly higher expressed in primed samples. The naïve marker gene *KLF17* was significantly and considerably enriched in naïve-like N2B27 and RSeT samples, respectively. *ZFP42* (also called *REX1*), which serves as a marker for naïve state pluripotency in mice but not humans^31^, was significantly higher expressed in both naïve as compared to primed culturing conditions. Furthermore, we found the cell surface marker *SUSD2*, which is a powerful tool to isolate and quantify human PSCs^32^, significantly upregulated in N2B27 but not RSeT naïve culturing conditions. Notably, the N2B27 protocol led to highest levels of naïve and PGC marker genes.

### NWR iPSCs contribute to tissues of developing mouse embryos

**Figure 4:**
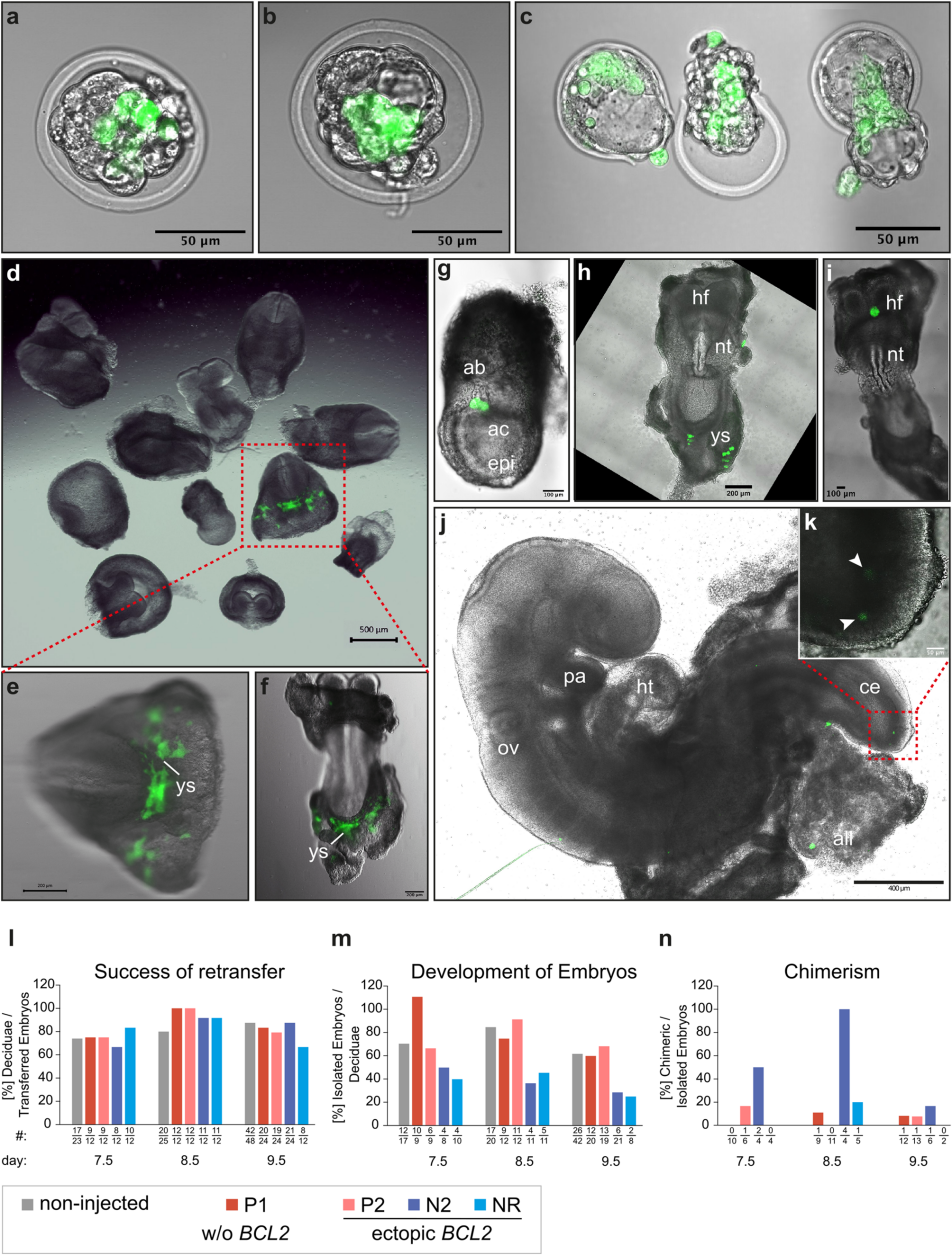
NWR iPSCs contribute to tissues of developing mouse embryos. See also Supplementary Table S2. **(a–c)** Photomicrographs of chimeric embryos generated by injection of NWR iBCL2-GFP-iPSCs into mouse blastocysts after 24 h (**a,b**) and 48 h (**c**) incubation in vitro. Hatching blastocysts were observed 48 h post injection (**c**). Scale bars: 50 µm. **(d–f)** Photomicrographs of chimeric embryos (developmental stage E8.5) generated by mouse blastocyst injections of primed stage NWR iBCL2-GFP-iPSCs followed by retransfer into pseudo-pregnant foster mice. Litter overview (**d**); posterior view (**e**); embryo outstretched, ventral view (**f**). Scale bars: 500 µm (**d**), 200 µm (**e,f**). **(g–k)** Photomicrographs of chimeric embryos generated by mouse blastocyst injections of naïve-like NWR iBCL2-GFP-iPSCs (N2B27 protocol), and retransfer into pseudo-pregnant foster mice at developmental stage E7.5 (**g**), E8.5 (**h,i**), and E9.5 (**j**,**k**). Ventral (**h**) and dorsal (**i**) view of outstretched specimen. Close-up of the caudal region of a 9.5 chimeric embryo (maximum intensity z-projection) depicts two GFP positive cells ((**k**), arrowheads). Scale bars: 400 µm (**j**), 200 µm (**h**), 100 µm (**g**,**i**), 50 µm (**k**). **(l)** Retransferred embryos successfully implanted into pseudo-pregnant foster mice. There was no significant difference between blastocysts injected with NWR iPSCs (P1, P2, N2 or NR culturing conditions) and non-injected control blastocysts. One-way ANOVA with Dunnett post-hoc correction. **(m)** The number of embryos, which can be isolated from the deciduae, provides information about the survival and development of implanted embryos. Comparison of injected (P1, P2, N2 or NR culturing conditions) and non-injected control blastocysts revealed no significant difference (one-way ANOVA with Dunnett post-hoc correction), however a tendency for less embryos derived from N2 and NR injected blastocysts was observed. **(n)** N2 culturing conditions led to an increased propensity to form chimeras. For statistical analysis, numbers of the three time points were summed up and all conditions compared with each other (all vs. all). The observed trend was not significant. Exact Fisher’s test with Holm–Bonferroni correction. **(l–n)** The number of samples is provided below the corresponding bar and in Supplementary Table S2. In one case (Figure 4m, P1, day 7.5) two embryos were isolated from one deciduae. *ab* allantoic bud, *ac* amniotic cavity, *all* allantois, *epi* epiblast, *ce* caudal end, *hf* headfold, *ht* heart tube, *nt* neural tube, *ov* otic vesicle, *ys* yolk sac, *P1* NWR iGFP-iPSCs, primed conditions, *P2* NWR iBCL2-GFP-iPSCs, primed conditions, *NR, N2* NWR iBCL2-GFP-iPSCs, naïve conditions, RSeT and N2B27 protocols, respectively. NWR iGFP-iPSCs have no ectopic *BCL2* expression, whereas in NWR iBCL2-GFP-iPSCs *BCL2* was introduced into the genome and ectopic expression was induced by doxycycline.

To investigate the potential of NWR iPSCs to contribute to tissues in vivo, we injected NWR iPSCs into mouse blastocysts. To identify NWR cells in chimeric embryos, we transduced NWR iPSCs with a doxycycline inducible GFP reporter construct (resulting in NWR iGFP-iPSCs), and treated the cells with doxycycline before injections. Culturing of injected mouse blastocysts revealed that in these interspecies chimera settings NWR iPSC became part of the inner cell mass (Fig. 4a–c), and that chimeric blastocysts hatched 48 h post injection normally (Fig. 4c). To gain insights into the further development of NWR-mouse chimeras, and to analyze the impact of the pluripotency state of the injected NWR iPSCs (naïve-like vs. primed), we retransferred injected and non-injected control blastocysts into pseudo-pregnant foster mice, which received doxycycline via their drinking water to sustain GFP expression in NWR iGFP-iPSCs and BCL2-GFP expression in NWR iBCL2-GFP-iPSCs during pregnancy (see “Methods” section, overview of injections in Supplementary Table T2). We isolated embryos at three developmental stages equivalent to embryonic day 7.5, 8.5 and 9.5, and analyzed the locations of GFP expressing cells. In all tested conditions, namely primed NWR iGFP-iPSCs (P1) and NWR iBCL2-GFP-iPSCs in primed (P2) as well as in naïve-like states (both N2B27, N2 and RSeT media, NR) we found NWR cells incorporated into tissues of the developing mouse embryo. In the majority of embryos, GFP positive cells were detected in extraembryonic tissues such as yolk sac (Fig. 4d–f,h), allantoic bud (Fig. 4g), allantois (Fig. 4j), and in a region around the headfold (Fig. 4i). The morphology of integrated GFP positive cells was similar to surrounding mouse cells indicating differentiation of NWR iPSCs and contribution to the host tissue. Especially in the yolk sac, we observed GFP positive cells with advanced morphology including cell processes (Fig. 4d–f,h). In one embryo derived from injections of naïve-like NWR iBCL2-GFP-iPSCs (N2 protocol) we detected GFP expressing cells also within the embryo proper at the caudal end (Fig. 4j,k).

We found no significant difference in the number of deciduae that formed after retransfer (Fig. 4l), and in the number of embryos that developed until the timepoints of isolation (Fig. 4m). However, we observed a tendency of less embryos that could be obtained from injections with naïve-like NWR iBCL2-GFP-iPSCs (both N2 and NR conditions, Fig. 4m). Consequently, the number of embryos that could be analyzed for GFP expressing NWR cells dropped for N2 and NR conditions. Comparison of the four conditions revealed no significant difference, but a higher tendency for N2 NWR cells to form chimeras (Fig. 4n).

Taken together, these results indicate that naïve N2B27 (N2) culturing conditions in combination with ectopic *BCL2* expression might not only improve the incorporation of NWR iPSCs into tissues of the mouse embryo proper, but also the formation of interspecies chimeras in general.

### Characterization of naïve-like pluripotency in NWR iPSCs

**Figure 5:**
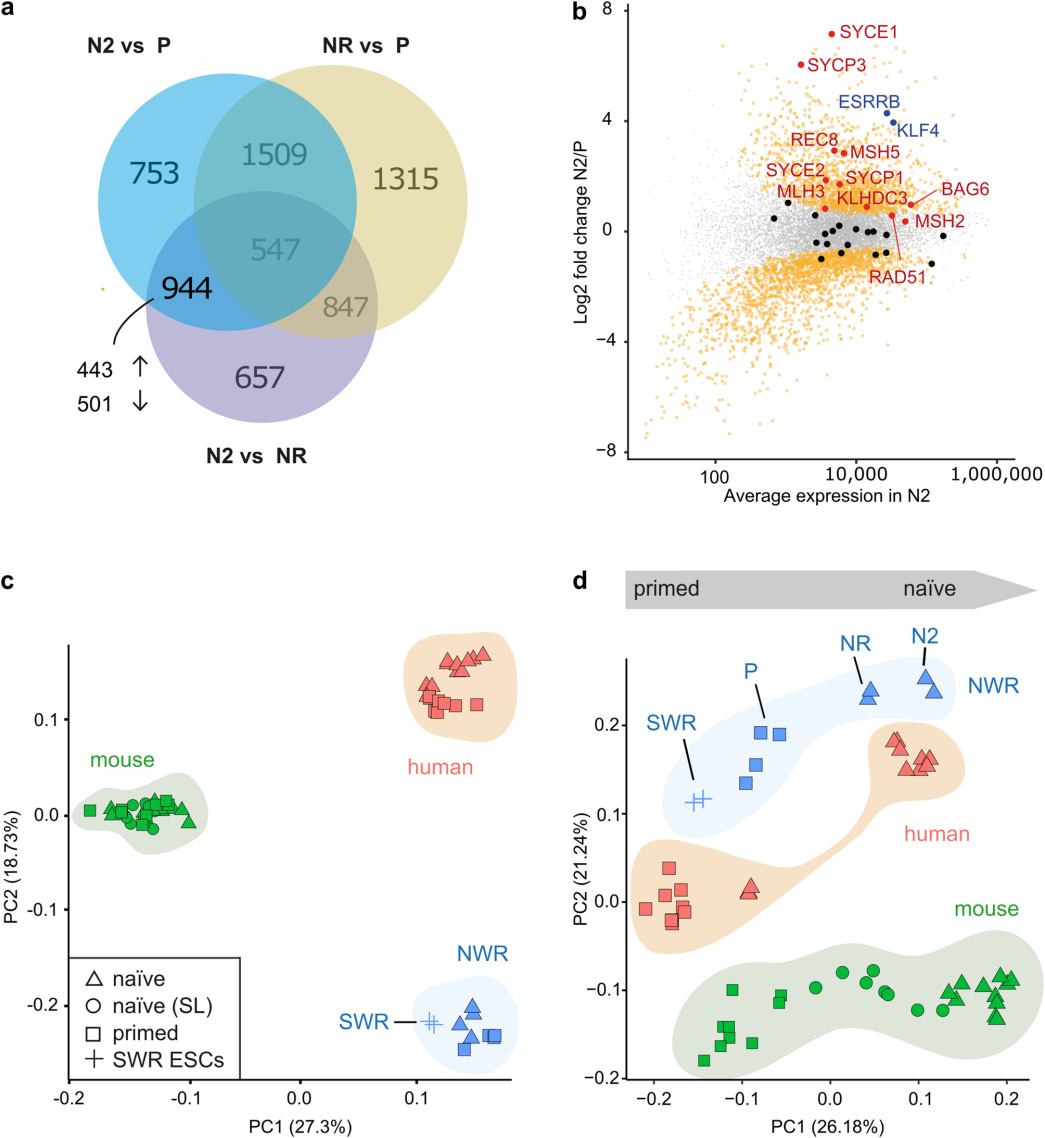
Comparative gene expression analysis. See also Supplementary Fig. S6, Supplementary Tables S1 and S3. **(a)** Venn-diagram depicting the number of genes, which significantly changed in the analyzed culturing conditions (DESeq2, Foldchange > 1.5, adjusted P value < 0.01). **(b)** Gene expression changes in naïve N2B27 (N2) as compared to naïve RSeT (NR) and primed mTeSR1 (P) samples. Plotted are log2fold changes N2 vs. P on y axis and the average gene expression in N2 culturing conditions on x axis. Orange: 944 genes, which specifically and significantly changed in N2. Blue: significantly enriched transcription factors known to regulate pluripotency in stem cells. Red: significantly enriched meiosis I related genes (GSEA analysis). Black and grey: meiosis I and all remaining genes, respectively, with no significant expression change. **(c,d)** Comparative gene expression analysis of NWR iPSCs with human, mouse and SWR ESCs cultured in naïve and primed conditions. Principal component analysis using all expressed genes **(c)** and a subset of 44 genes, which were detected in all analyzed species, and associated with housekeeping, pluripotency, stemness, naïve and primed state (**(d)**, see Supplementary Table S1 for details). *P* NWR iPSCs primed conditions (mTeSR1), *NR, N2* NWR iBCL2-GFP-iPSCs, naïve conditions, RSeT and N2B27 protocols, respectively, *naïve (SL)* mouse ESCs cultured in serum-LIF conditions.

Better engrafting efficiency in mouse blastocysts (Fig. 4n) and higher expression of naïve marker genes in NWR iBCL2-GFP-iPSCs cultured in naïve N2B27 medium (Fig. 3c,d), prompted us to look closer at genes specifically expressed in these cells. Therefore, we performed differential gene expression analysis between all samples of the transcriptomics dataset (naïve N2B27 (N2) vs. primed (P); N2 vs. naïve RSeT (NR); NR vs. P), and focused on the intercept of N2 vs. P and N2 vs. NR (Fig. 5a). In total, we identified 944 genes, which were exclusively differentially expressed in naïve N2B27 conditions in these comparisons. Out of those, 443 and 501 genes were up- and down-regulated, respectively. Interestingly, we found *KLF4* and *ESRRB*, genes that are directly involved in establishment and maintenance of naïve pluripotency (reviewed in Ref.^33^), in the significantly up-regulated genes of the naïve-like N2B27 samples (Fig. 5b, marked in blue). Notably, the expression of the integrated exogenous human *KLF4* (*hKLF*) changed comparably little between culturing conditions (endogenous NWR *KLF4* and *hKLF4* 17.3-fold and 2.8-fold increased, respectively, in naïve N2B27 as compared to primed conditions, Supplementary Fig. S6b). As Endogenous NWR *KLF4* is regulated by its endogenous promoter*,* whereas exogenous *hKLF4* is driven by the coMIP247 encoded spleen focus-forming virus (SFFV) promoter, which is not responsive to different culturing modalities, a much stronger response of endogenous NWR *KLF4* to the applied culturing conditions was expected and highlights that the core pluripotency regulatory network is not obviously affected by the (overall low) expression of the integrated reprogramming factors.

To evaluate reactivation of the X chromosome in naïve-like NWR iPSCs, we measured the expression of the X-inactive specific transcript (*XIST*), a long non-coding RNA which specifically silences one X chromosome in female cells. We found substantial lower expression in N2B27 samples (Supplementary Fig. S6d), indicating two active X Chromosomes in naïve-like NWR iPSCs. Additionally, using gene set enrichment analysis (GSEA), we found meiosis I related genes significantly overrepresented among the N2B27 upregulated genes (Fig. 5b, 11 out of 20 meiosis I related genes (GO:0007127) significantly enriched, false discovery rate P value 0.00932, marked in red). Comparison of N2 and P revealed “metabolic pathways” and “oxidative phosphorylation” as the top significantly upregulated KEGG pathways in naïve-like N2B27 NWR iPSCs (P values 9.407292 × 10^−07^ and 1.204395 × 10^−04^, respectively; Supplementary Fig. S6c).

### Comparative gene expression analysis of NWR iPSCs with human, mouse and SWR ESCs

To compare the expression profiles of NWR iPSCs with respective transcriptomes from human and mouse ESCs, we combined our data with previously published RNA-sequencing datasets (Supplementary Table S3). Furthermore, we added unpublished transcriptome data from SWR ESCs^9^. PCA with all expressed genes as input separated the samples by species [human, mouse and white rhinoceros (WR)], indicating a higher variance between species than pluripotency states (Fig. 5c). PC1 (explaining 27.30% of the variance) separated human/WR from mouse, while PC2 (explaining 18.73% of the variance) separated human/mouse from WR, indicating higher similarity between WR and human than WR and mouse transcriptomes. Next, we focused on a set of 46 marker genes, which were detected in all analyzed species, and which are associated with naïve and primed states as well as stemness, pluripotency and housekeeping (see Supplementary Table T1 for details). PCA with this subset as input clearly separated the pluripotency states along the PC1 axis (explaining 26.18% of the variance). Notably, SWR ESCs showed a higher degree of variance from naïve-like than primed NWR iPSCs with greatest distance to the naïve N2B27 protocol, whereas NWR iPSCs and human ESCs (both in respective naïve states) localized close to each other (Fig. 5d). This indicates that NWR iPSC can be converted into a naïve-like state of pluripotency, which has a similar expression profile of pluripotency and stemness related genes as naïve state human ESCs.

## Discussion

The importance of advanced assisted reproduction technologies (aART) may go beyond supporting conventional conservation approaches such as habitat protection and breeding as they might in some cases be the last resort to rescue critically endangered species from extinction. With just two females remaining, the NWR symbolizes the ‘catastrophic decline’ in flora and fauna, and especially of large mammals, which happens globally due to human activities^1–3,34^. Nevertheless, there is hope that biomedical technologies could be applied to rescue keystone species from extinction, or at least to produce cell banks for future resurrection when technologies become available^5^.

In this context, it is crucial to prepare grounds for stem cell differentiation to functional gametes^35^ by expanding the knowledge about the regulation of PSCs, pluripotency and differentiation, without having access to embryos of the endangered species itself. Reprogramming factors can be used to produce iPSCs from adult somatic cells such as skin fibroblasts^36^, and the generation of iPSCs from NWR samples has been shown previously^10,11^.

Here, we successfully applied a virus-independent reprogramming strategy using episomal plasmids in combination with feeder-free culturing conditions to produce NWR iPSCs from the deceased female Nabire. We analyzed the karyotype of NWR iPSCs and the corresponding source fibroblasts and detected 2n = 81 chromosomes including two X and one marker chromosome. Although, the karyotype of most rhinoceros species (4/5 analyzed) compromises usually 2n = 82 (white, Indian, and Sumatran rhinoceros) or 2n = 84 (black rhinoceros) chromosomes^37^, a complement of 2n = 81 has been described previously in the wild-born male Sudan (Stud# 372) and his daughters Nabire (Stud# 789) and Najin (Stud# 943), which have been born in captivity^22^. In the same study, a metacentric chromosome (here labeled as marker chromosome) has been reported. The loss of one chromosome might be the result of a Robertsonian translocation, in which one chromosome gets attached to another. This type of chromosomal abnormality usually does not affect health, but has been connected to infertility in humans^38^. As both Sudan and Najin successfully bred in captivity, although reproduction of pure NWRs in zoos overall has been low, the production of gametes is not impaired due to a karyotype of 2n = 81 chromosomes, and might reflect a natural genetic variance in NWRs.

The integration of one out of two used reprogramming vectors into the genome of NWR iPSCs renders the cells generated in this study unsuitable for in future gamete and embryo production. However, as the expression of plasmid encoded genes was very low on RNA, and at the detection limit on protein level, we assume that pluripotency in the generated NWR iPSCs is—unlike in PSCs derived from other animal species such as pig^39^—maintained independently from transgene expression, but controlled by expression of endogenous factors, thereby making these cells a valuable tool to investigate the regulation of pluripotency and differentiation of rhinoceros stem cells. We performed unprecedented global transcriptomics of white rhino (WR) PSCs and improved gene annotation of the WR transcriptome. Gene expression comparison of NWR iPSCs with human, mouse and SWR ESCs revealed high similarity of primed NWR iPSCs and SWR ESCs indicating that reprogramming induced a gene expression pattern that resembles natural rhinoceros PSCs. Cultured in medium without inhibitors, SWR ESCs reside in primed state pluripotency alike human ESCs. Along this line, NWR iPSCs morphologically resembled primed state human ESCs and iPSCs, and readily reacted to human differentiation protocols thereby giving rise to cells of the three germ layers as well as trophoblast. As most effective protocols for PGC differentiation necessitate PSCs at a naïve state of pluripotency (reviewed in Refs.^12,13^), we tested two naïve culturing conditions established for human PSCs (based on Refs.^26,27^) on NWR iPSCs. Ectopic expression of *BCL2* assisted the conversion of cells into putative naïve states by inhibiting apoptosis. *BCL2* has also been reported to improve the formation of interspecies chimeras^40^. However, in our blastocyst injection experiments, ectopic expression of *BCL2* alone did not improve engraftment of NWR iPSCs into mouse embryos (no difference observed between non-*BCL2* overexpressing NWR iPSCs-iGFP (primed, P1 conditions) and *BCL2*-overexpressing NWR iPSCs-iBCL2-GFP (primed, P2 and naïve RSeT, NR conditions), Fig. 4n). Just the combination of ectopic *BCL2* within the context of the naïve N2B27 protocol led to an increase in the number of embryos containing GFP positive NWR cells indicating higher impact of culturing conditions than transgene overexpression. We observed a tendency for reduced development of blastocysts injected with naïve-like NWR iBCL2-GFP-iPSC [both N2B27 (N2) and RSeT (NR) conditions]. As a faster cell doubling time and improved survival has been reported for PSCs in naïve state pluripotency^41^, we speculate an imbalance of NWR and mouse cells within N2 injected embryos, which hampered their development and thus lowered the number of embryos that could be isolated and subsequently analyzed. As only embryos with a correct ratio of NWR and mouse cells could survive, the likelihood for a blastocyst injected with N2 NWR iPSCs to be chimeric was increased. Given the wide evolutionary distance of mouse and NWR, interference of NWR iPSCs with mouse embryonic development came not as a surprise. The capacity to generate full interspecies chimeras depends on multiple factors such as the extracellular environment (including signaling molecules, ligands, and/or adhesive molecules), cell proliferation rate, and developmental timing (reviewed in Ref.^42^). It has been shown that human PSCs can contribute to both human-mouse^26,43^ and human-porcine chimeras^44^, albeit at very low rates. Another study reported that, unlike rodent PSCs, human PSCs only contributed to extraembryonic tissues after injection into mouse embryos, but never to the embryo itself^45^. Comparably, we detected NWR iPSCs predominantly (11/12 analyzed chimeras) in extraembryonic tissues and just once within the embryo proper. The ability of NWR iPSCs to contribute to tissues of mouse embryos at early stages of development indicates high developmental potency. However, to better understand the cellular potential of NWR iPSCs in vivo, a suitable host species and environment has to be identified. Given the rather close relationship, horse blastocysts might be a good option.

Most interspecies chimeras (including one embryo with incorporation of NWR iPSCs into the embryo proper) were generated from injections of naïve-like NWR iBCL2-GFP-iPSCs cultured in N2B27 medium. Gene expression comparison of naïve-like and primed state NWR iPSCs revealed highest expression of naïve marker genes and induction of the transcription factors *KLF4* and *ESRRB*, which directly support naïve pluripotency (reviewed in Ref.^33^), specifically in N2B27 conditions. Additionally, we found *DNMT3B* and *XIST* significantly and substantially lower expressed in N2B27 culturing conditions, respectively. *DNMT3B* is the major DNA methyltransferase expressed and active at early stages of embryonic development, and low expression levels have been associated with genome-wide DNA hypomethylation and epigenetic erasure, which are both characteristics of naïve state pluripotency^46^. A particular epigenetic hallmark associated with naïve pluripotency is the reactivation of the X chromosome. In female cells, the long non-coding RNA *XIST* is solely expressed from the inactive X chromosomes (reviewed in Ref.^47^). Therefore, lower *XIST* expression indicates two active X chromosomes in naïve-like N2B27 NWR iPSCs. Furthermore, a switch in metabolism from glycolysis in primed PSCs to both glycolysis and oxidative phosphorylation in naïve PSCs has been reported^48^. We found “metabolic pathways” and “oxidative phosphorylation” as the two top-enriched KEGG pathways in N2B27 as compared to primed samples, which provides additional support that NWR iPSCs cultured in N2B27 conditions reside in a naïve-like state of pluripotency. Similar to PGCs^49^, PGC marker and meiosis I related genes were induced in naïve-like N2B27 NWR iPSCs.

The identified molecular attributes together with the observed colony morphology and chimerism propensity suggest that N2B27 conditions (unlike the RSeT protocol) introduce a naïve-like state, which represents pluripotency in the rhinoceros embryo. The gained knowledge about pluripotency in the order odd-toed ungulate (Perissodactyla) provides the ground for adapting human differentiation protocols towards generating NWR gametes in vitro, which could potentially help to rescue the species from extinction.

## Methods

### Derivation of NWR primary fibroblasts

The female NWR Nabire was housed in ZOO Dvůr Králové, Czech Republic. A skin biopsy was taken under general anaesthesia in the Regio axillaris. The Biopsy area was widely disinfected with Octenosept Spray (TM, SCHÜLKE & MAYR GmbH, #121411) and the deep skin biopsy was achieved by using a Kai Biopsy Punch device (KAI Medical, 4 mm in diameter, # BP-40F), sterile surgical forceps and a sterile scalpel. The recovered tissue was immediately transferred into cell culture medium (DMEM, ThermoFisher, #41965039) supplemented with 1× Antibiotic–Antimycotic (Sigma-Aldrich, #A5955) and shipped at 4 °C. The wound area was sutured with simple surgical suture using 3/0 seam material with a sharp needle (Supramid®, 3/0 HS23—0.45m B. Braun Petzold, #C0712256). The wound was covered against flies and better wound healing with veterinary aluminum wound spray (Pharmamedico GmbH, #03691157).

Upon arrival in the cell culture lab, the skin biopsy was diced into small pieces, and subsequently incubated with 2 mg/ml Collagenase Type IV solution for 30 min at 37 °C. Collagenase solution was prepared by dissolving Collagenase Type IV powder in DMEM/F12 medium (ThermoFisher, #17104019 and #31331093). After the enzymatic dissociation, the tissue was minced with forceps and dissociated further by pipetting. Cells were derived using a 1:1 mix of FibroGRO (Merck Millipore, #SCM037) and Advanced MEM (ThermoFisher, #12491015). The latter was supplemented with 5% HyClone Fetal Bovine Serum (FBS, GE Healthcare, #SV30160.03HI), 1× MEM NEAA and 1× GlutaMAX (ThermoFisher, #11140050 and #35050061). The cells were plated on plates pre-coated with 0.2% Gelatin solution (AppliChem, #A1693) supplemented with 1% HyClone FBS. Glass coverslips were placed on the tissue clumps to ensure their attachment. The cultures were supplemented with 2% Penicillin–Streptomycin and 1:1000 dilution from Gibco Amphotericin B (ThermoFisher, #15140122 and #15290026), and grown at 37 °C with 5% CO_2_ and 5% O_2_ for five days. When cultures reached ~ 90% confluency (day 9–12), they were passaged by trypsinization into single cells using 0.25% Trypsin–EDTA (ThermoFisher, #25200056), replated at a density of 2.5 × 10^5^ cells/100 mm tissue culture dish, and grown in Advanced MEM as described above. Fibroblasts (2 × 10^6^ cells/vial) were cryopreserved using Bambanker freezing medium (NIPPON Genetics EUROPE, #BB01).

### Reprogramming of NWR fibroblasts

NWR fibroblasts were trypsinized and 1.5 × 10^6^ cells transfected using MEF 1 Nucleofector Kit solution (Lonza, #VPD-1004) combined with a total of 12 µg plasmid DNA [9 µg MIP 247 CoMiP 4in1 harboring IRES-dTomato with p53 shRNA U6 cassette (short coMIP247) and 3 µg pCXLE-hMLN; Addgene, #63726 and #27079]. The cells were pulsed with the T-020 program using the Nucleofector 2b Device (Lonza, #AAB-1001). Transfected cells were plated onto Matrigel coated plates. The Matrigel coating solution was prepared using a 1:100 dilution of 1 ml Matrigel (Corning, #354234) in DMEM/F12 (ThermoFisher, #17104019). For the first 24 h, cells were fed with the Advanced MEM formulation used to grow NWR fibroblasts. Subsequently, the culture was switched to M15 reprogramming medium containing 20% KnockOut Serum Replacement (ThermoFisher, #10828028) and 60 µL human LIF (Milipore, #LIF1010)^21^. After the initial morphological changes (~ day 15), cultures were switched to three different pluripotency supporting media: bFGF medium, mTeSR1 medium (StemCell Technologies, #05850), and 2i-hLIF medium. The bFGF medium formulation consisted of DMEM/F12, 20% KnockOut Serum Replacement, 10 ng/ml bFGF (Peprotech, #100-18B), 1× MEM NEAA, 1× GlutaMAX, and 0.5% 2-Mercaptoethanol (ThermoFisher, #31350010). The 2i-hLIF media formulation consisted of KnockOut DMEM (ThermoFisher, #10829018), 20% KnockOut Serum Replacement, 60 µl human LIF, 5 µM CHIR99021 and 1 µM PD0325901 (TOCRIS, #4953 and #4192). Based on morphological assessment of the reprogramming cultures, colonies were manually picked on day 21. Only mTeSR1 enabled establishment of NWR iPSC lines. Three colonies were expanded further and cryopreserved. Subsequent analysis was mainly performed with 2/3 generated NWR iPSC lines.

### Generation of NWR iBCL2-GFP-iPSCs via nucleofection

The backbone of PB-FLAG-LIN28B-T2A-GFP (gift from Yoav Mayshar, Harvard University), was amplified by PCR with primers that exclude LIN28B-T2A-GFP sequence. The coding sequence of eGFP was amplified separately from the same vector. The coding sequence of human *BCL2* (transcript variant α, NM_000633) was PCR amplified from cDNA prepared from the human ESC line H9. All PCR fragments had 30-bp overlaps to the adjacent vector fragments. The P2A bridge with GSG linker (ggatccggagccacgaacttctctctgttaaagcaagcaggagacgtggaagaaaaccccggtccc) was part of the PCR primers. Final vector was assembled from column-purified PCR fragments using Gibson assembly master mix (NEB, #E2611L) according to the manufacturer’s instructions. Correct assembly was verified by Sanger sequencing. 3 µg of the PB-FLAG-BCL2-P2A-GFP vector mixed with 3 µg of a vector coding for Piggybac transposase were transfected into NWR iPSCs via nucleofection using P3 Primary Cell 4D-Nucleofection X Kit (Lonza, #V4XP-3024) and 4D-Nucleofector System (Lonza), according to the protocol recommended by the manufacturer. Nucleofected cells were grown in mTeSR1 supplemented with 50 µg/ml Hygromycin B (ThermoFisher, #10687010) for 1 week to select cells with stable genomic integration. After picking, subclones were routinely maintained with 25 µg/ml Hygromycin B and 1 µg/ml doxycycline (Clontech, #631311) to induce *BCL2* and GFP expression. Subsequent experiments were performed with one subclone.

### Lentiviral generation of NWR iGFP-iPSCs

To generate an inducible NWR iPSC reporter line, we transduced NWR iPSCs with the custom-made lentiviruses Lenti-GFP and Lenti-rttA^50^. Therefore, we plated 5 × 10^4^ NWR iPSCs on a Matrigel coated 24-well in mTeSR1 medium supplemented with ascorbic acid (50 µg/ml) two days before transduction. On the day of transduction, 15 µl of each virus were mixed with polybrene (final concentration 7 ng/µl, Merck Millipore, #TR-1003-G) in 500 µl mTeSR1 medium supplemented with ascorbic acid. Twenty-four hours after incubation in the virus mixture, NWR iPSCs were washed once with 1 ml medium and cultured further in mTeSR1 supplemented with ascorbic acid and doxycycline (2 µg/ml, Sigma-Aldrich, #D3447). To deplete for non-transduced cells, puromycin selection (0.8 µg/ml, ThermoFisher, #A11138-03) was started on the next day for 5 days. Medium was changed and doxycycline as well as puromycin were added fresh daily. On day 7 after transduction, NWR iPSCs were split with PBS 0.5 mM EDTA on a Matrigel coated 10 cm cell culture dish and fed with mTeSR1 medium supplement with ascorbic acid and doxycycline. On day 16 after transduction, several GFP positive colonies were isolated and propagated individually in Matrigel coated 24-wells in mTeSR1 medium supplement with ascorbic acid and doxycycline. Colonies displaying a strong GFP signal after two passages were cryopreserved until further use.

### Culture conditions

#### Primed culturing conditions

NWR iPSCs were maintained feeder-free on Matrigel coated 6-well plates in chemically defined mTeSR1 medium (STEMCELL technologies, #85850) supplemented with ascorbic acid (50 µg/ml, Sigma-Aldrich, #A8960-5G) at 37 °C, 5% CO_2_ and 5% O_2_. The human iPSC line BIHi005A was maintained in homemade, chemically defined E8 medium [DMEM/F12 HEPES (ThermoFisher, #11330032) supplemented with l-Ascorbic acid 2-phosphate (Sigma, #A8960), Insulin (CS Bio, #C9212-1G or Sigma, #91077C-1G), Transferrin human (Sigma,#T3705-1G), Sodium Selenite (Sigma, #S5261-10G), bFGF (PeproTech, #100-18B), TGFβ1 (PeproTech, #100-21C) and Sodium Bicarbonate 7.5% solution (Fisher Scientific, #25,080–094), according to Ref.^51^]. Cells were split every 3 to 4 days (confluency ~ 80%) using PBS 0.5 mM EDTA solution (Life technologies, #14190-250 and ThermoFisher, #15575-020) in ratios of 1:6 and 1:12. ROCK inhibitor (Y-27632 2HCl; 10 mM/1 ml, Selleck Chemicals, #SEL-S1049-10MM) was added to the medium at 10 µM for the first 24 h after passaging.

#### Naïve culturing conditions

To induce the naïve state, cells were transitioned from primed conditions either to RSeT (Stemcell Technologies, #05970) or N2B27 medium [1:1 DMEM/F12: Neurobasal medium (#11320-033 and #21103-049), 1× N2-Supplement (#17502-048), 1× B27-Supplement w/o Vit. A (#12587-010), 1× GlutaMAX (#35050-038), 1× MEM NEAA (#11140-035), 0.11 mM ß-Mercaptoethanol (#21985-023), 10 ng/ml LIF (Merck/Millipore, #LIF1010), 0.075 µM Bio (Tocris Bioscience, #3194), 5 µM XAV (Tocris Bioscience, #3748), 0.5 µM PD-0325901 (BioVision, #1643), 2 µM Gö6983 (Tocris Bioscience, #6983), 75 µg/ml ascorbic acid (Sigma-Aldrich, #A8960), 15 ng/ml Activin A (#PHC9564). If not indicated otherwise, all medium components were from Life Technologies] as follows: After TrypLE split (TrypLE 1x, Life Technologies, #12563-011), primed iPSCs were plated as single cells in ratios of 1:6 and 1:12 on 5.0 × 10^5^ MEFs/6-well (mitomycin C-treated mouse embryonic fibroblasts, teBu-bio, #003MEF-MITC) with 10 µM ROCK inhibitor (Y-27632 2HCl; 10 mM/1 ml, Selleck Chemicals, #SEL-S1049-10MM) and incubated overnight in mTeSR1 (NWR iPSCs) or E8 medium (human BIHi005A). At the next day, medium was changed to N2B27 or RSeT. To facilitate the conversion process, the NWR iBCL2-GFP-iPSC line was generated (see above) and ectopic expression of *BCL2* was induced by 1 µg/ml doxycycline (Clontech, #631311). Cells were maintained on MEFs in N2B27 or rather RSeT medium at 37 °C, 5% CO_2_ and 5% O_2_, and split using TrypLE every 3 to 4 days (confluency ~ 80%) in ratios varying from 1:4 to 1:10. ROCK inhibitor was added to the medium at 10 µM for the first 24 h after passaging.

#### SWR ESC culturing conditions

SWR ESCs [051B line^9^] were maintained in DK20 [DMEM/F12 (Gibco, #11320033) supplemented with 20% (vol/vol) KSR (Gibco, #10828028), 1× MEM NEAA (Gibco, #11140050), 1× GlutaMAX (Gibco, #35050061), 0.1 mM 2-Mercaptoethanol (Gibco, #21985023), 50 U/ml penicillin/streptomycin (Gibco, 1570063), 10 ng/ml recombinant human bFGF (Wako, #064-04541)], and passaged every 4 to 5 days using TrypLE (Gibco, #12604021). SWR ESCs were routinely plated at a density of 1 × 10^5^ cells on one well of a 6-well plate laid with 2.0 × 10^5^ MEFs. ROCK inhibitor (Y-27632; Wako, #03424024) was added to the medium at 10 µM for the first 24 h after passaging.

### G-banding of NWR iPSCs

Karyotyping of NWR skin fibroblasts and iPSCs was performed by the Laboratory for Human Genetics in Berlin. In brief, metaphase chromosomes were obtained from NWR cells following a standard protocol for monolayer cultures and by treating the cells overnight with colcemid. Thereafter, G-banding of single mitotic cells was performed. Karyograms were documented with the Axio Imager Z2 from Zeiss 630× and assembled using the Metafer and Ikaros software from MetaSystems.

### In vitro differentiation of the three germ layers

Endoderm was differentiated from NWR iPSCs using the STEMdiff Definitive Endoderm Kit (Stemcell Technologies, #05110) following the manufacturer’s instructions. In brief, on day 0, cells with 70–80% confluence were dissociated using Accutase (Life technologies, #A1110501) and plated at a density of 2.1 × 10^5^ cells/cm^2^ in mTeSR1 medium with 10 µM ROCK inhibitor (Y-27632, SelleckChem, #S1049). On day 1, the medium was changed to STEMdiff Endoderm Basal Medium 1. On day 2, the medium was replaced with STEMdiff Endoderm Basal Medium 2 until day 4 with medium changes every day. On day 5, the cells were fixed and analyzed by immunostaining.

The neural (ectodermal) differentiation of NWR iPSCs was performed using a modified version of the protocol published by Chambers et al.^52^. In brief, 2 × 10^5^ NWR iPSCs were seeded into one well of a Geltrex coated 6-well plate using mTeSR1 and 10 µM ROCK inhibitor. Thereafter, the medium was changed daily for 3 days using plain mTeSR1. Afterwards, the medium was replaced with neural differentiation medium [500 ml DMEM/F12 (ThermoFisher, #12660-012) mixed with 10 ml B27 (ThermoFisher, #17504-044) and 5 ml N2 (ThermoFisher, #17502-048) supplemented with 10 µM SB431542 (Reagents Direct, #21-A94) and 2 µM Dorsomorphin (Biovision, #1686-5)]. The neural differentiation medium was replaced daily for 10 days. In addition, we used the protocol described by Pak et al.^53^ to induce functional neurons (iNeurons) from NWR iPSCs.

Induction of the mesoderm differentiation was performed as described in Burridge et al., 2014^54^. In brief, 4 × 10^5^ NWR iPSCs were seeded as single cells supplemented with 10 µM ROCK inhibitor on one well of a Geltrex (Life Technologies, #A1413202) coated 6-well plate in mTeSR1 (R&D System, #AR005) for 3–4 days until the cells were 90–100% confluent. Thereafter, the medium was changed to RPMI medium 1640 (ThermoFisher, #21875091) containing CDM3 supplement [recombinant human albumin (Sigma, #A0237), l-ascorbic acid 2-phosphate (Sigma, #A8960)], and the GSK3 inhibitor CHIR99021 (Stemcell Technologies, #72054, 6 µM)] in order to activate the WNT signaling pathway and thereby the cardiac differentiation of the cells. Two days later, the medium was replaced with RPMI medium 1640 with CDM3 supplemented with 5 µM IWP2 (Stemcell Technologies, #72122). Beating cells were observed earliest 10 days later as small clusters.

### Trophoblast progenitor differentiation

NWR iPSCs were differentiated towards trophoblast progenitors as described previously^24^. Briefly, colonies were dissociated with Accutase (Sigma, #A6964) and seeded as single cell monolayers (1.05 × 10^5^ cells/cm^2^) into Matrigel-coated 8-well chamber slides (Ibidi, #80826) with KSR-based differentiation medium [DMEM/F12 (#31331093), supplemented with 20% KSR (#10828028), 1× GlutaMAX (#35050061), 1× MEM NEAA (#11140050) and 1% Penicillin–Streptomycin (#10378016). All medium components were from ThermoFisher], supplemented with 50 ng/ml BMP4 (R&D systems, #314-BP). Fresh medium with BMP4 was applied every 24 h for a total of 72 h.

### Immunofluorescence

Primary and secondary antibodies used for immunostaining are listed in Supplementary Table T4.

To check the expression of pluripotency and trophoblast markers, NWR iPSCs were grown on Matrigel-coated ibidi 8-well chamber slides (ibidi, #80826), and fixed either directly (to address pluripotency) or after trophoblast progenitor differentiation (see above) with 4% PFA/DPBS solution (Pierce 16% Formaldehyde (w/v), Methanol-free, ThermoFisher, #28906) for 15 min at room temperature. Subsequently, cells were permeabilized with 0.2% TritonX-100/DPBS (Sigma Aldrich, X100-500ML) solution for 15 min at room temperature. Primary and secondary antibodies were diluted in 10% FBS/0.2% TritonX-100/DPBS and incubated overnight at 4 °C and 1 h at room temperature, respectively.

For immunostaining against dTOMATO, NWR iPSCs were grown on Geltrex coated 8-well chamber slides (Falcon, #654108), fixed with BD Cytofix (BD Biosciences, #554655) for 15 min at room temperature, and subsequently permeabilized and blocked in NDB solution [0.4% TritonX-100 (Sigma, #T8787), 0.2% BSA (Biomol, #1400100), 10% normal donkey serum (abcam, #ab7475) in DPBS] for 1 h at room temperature. Primary antibodies were diluted in NDB solution and incubated over night at 4 °C. Secondary antibodies were diluted in DPBS and incubated for 1.5 h at room temperature.

To address the potential of NWR iPSCs to differentiate into the three germ layers, NWR iPSCs were differentiated in 24 well plates to cardiomyocytes (mesoderm), primitive endoderm cells and neural stem cells (ectoderm) as described above. Subsequently, cells were fixed, permeabilized, blocked and stained using the Human Cardiomyocyte Immunocytochemistry Kit (ThermoFisher, #A25973) according to the manufacturer’s instructions.

DAPI (50 µg/ml) solution was used for nuclei staining. Imaging of pluripotency and trophoblast markers was performed using a Zeiss Axiovert 200M epifluorescent microscope. dTOMATO and markers of the three germ layers were analyzed using the LEICA DIMi8 microscope and LAS X Software. Cardiomyocytes were additionally imaged using an LSM 510 Meta inverted confocal microscope (Carl Zeiss) and the ZEN software (Carl Zeiss).

### Generation and analysis of NWR-mouse interspecies chimeras

Interspecies chimeras were generated by injecting mouse blastocysts with either iGFP or iBCL2-GFP expressing NWR iPSCs cultured in primed or naïve conditions (overview in Supplementary Table T2) according to standard procedures^55^. For blastocyst injections approximately 15 iPSCs were injected into C57BL/6NCrl host blastocysts. In vitro culture of embryos was performed in microdrop culture under mineral oil (Sigma, #M8410) in KSOM medium at 37 °C with 5% CO_2_.

Chimeric blastocysts (and non-injected control blastocysts) were retransferred on the day of injection unilaterally into the uterus of Hsd:ICR (CD-1) pseudo-pregnant foster mothers at day 2.5 after plug. When necessary, the transgene expression was sustained by addition of doxycycline (5 µg/ml) into the embryo culture media and by supplementing doxycycline (4 mg/ml) to the drinking water of the foster animals during pregnancy. To cover the taste of doxycycline, 6% saccharose was added to the drinking water. Embryos were isolated from the foster animals at day 5, 6 and 7 after retransfer, to obtain the developmental stages E7.5, E8.5 and E9.5 respectively, and analyzed for GFP expressing cells. Images were taken with a Zeiss Axiozoom.V16 Fluorescence Stereo Zoom Microscope (Carl Zeiss MicroImaging) or a Zeiss LSM710NLO (Carl Zeiss MicroImaging) and processed with the Zeiss ZEN software package (Carl Zeiss MicroImaging). For statistical analysis, GraphPad Prism software (v9.2.0) was used. To test, if there is a significant difference in retransfer efficiency (number of deciduae/number of retransferred embryos) and survival of embryos (number of isolated embryos/number of deciduae) we compared each condition (P1, P2, N2, NR) individually with non-injected control blastocysts using one-way ANOVA with Dunnett post-hoc correction. To compare all culturing conditions with each other (all vs. all) in regards to the number of observed chimeras (number of chimeras/number of isolated embryos), we summed values across the three time points and performed Exact Fisher’s test with Holm-Bonferroni correction. All animal procedures were approved by the local authority (LAGeSo, Berlin) and performed under the license G0200/12-43 in accordance with relevant guidelines and regulations.

### PCR to test for integration of reprogramming vectors

To test for integration and expression of the reprogramming vectors coMIP247 and pCXLE-hMLN in NWR iPSCs, both genomic DNA (gDNA) and RNA were isolated and analyzed by PCR. Therefore, NWR iPSCs cultured in mTeSR1 on Geltrex coated 6-well plates were harvested using TrypLE. For gDNA, cell pellets from one 6-well were stored at − 20 °C until further use. For RNA, cell pellets from one 6-well were resuspended in RLT buffer (RNeasy Mini Kit, Qiagen, #74106) and stored at − 80 °C until further use. gDNA was extracted using the DNeasy Blood & Tissue Kit (Qiagen, #69506) following the manufacturer’s instructions. During extraction, the proteinase K digested cell suspension was treated with RNase A (20 µg/ml, Promega, #A797A) for 15 min at 37 °C to ensure RNA-free gDNA. For RNA extraction, cell lysates were processed according to the manufacturer’s protocol. RNA concentration was measured by nanodrop and 1 µg RNA was digested with DNase (RQ1 RNase-free DNAse, Promega, #M6101) at 37 °C for 30 min to remove potential contaminating DNA molecules. The reaction was terminated by adding 1 µl RQ1 DNase Stop Solution and incubation at 65 °C for 10 min. Subsequently, 200 ng DNase treated RNA was reverse transcribed into cDNA using random hexamer primer and the Superscript III kit (ThermoFisher, #18080051) according to the manufacturer’s instructions in a reaction volume of 20 µl. As negative control, 200 ng DNase treated RNA was processed in parallel, but without adding reverse transcriptase (− RT control). In the end, the cDNA was diluted 1:5 with water (final cDNA volume 100 µl).

PCR was performed using the AmpliTaq Gold 360 Master Mix (ThermoFisher, #4398901) in a 25 µl reaction volume. As template, 200 ng gDNA, 3 µl cDNA (both + RT, and − RT control), 30 pg of either coMIP247 or pCXLE-hMLN reprogramming vector (positive control) were used. Primers (see Supplementary Table T5) were used at a final concentration of 0.5 µM. DNA template was amplified in 35 PCR cycles. The annealing temperature was set to 58 °C. As control, the endogenous NWR *NANOG* gene was amplified with exon spanning primers to monitor potential RNA or rather DNA contamination in gDNA and cDNA, respectively.

### Validation of marker gene expression by RT-qPCR

RNA was extracted from NWR iBCL2-GFP-iPSCs cultured in primed (mTeSR1) or naïve (N2B27 and RSeT) conditions, DNase digested and transcribed into cDNA as described in the previous paragraph. qPCR was performed using QuantiTect SYBR Green PCR Kit (1000) (Qiagen, #204145) and a QuantStudio 6 Flex Real-Time PCR System (ThermoFisher, #4485691). Primers (see Supplementary Table T5) were used at a final concentration of 0.5 µM. For reagent activation and initial denaturation, samples were incubated at 50 °C for 2 min followed by 7 min at 95 °C. Subsequently, cDNA was amplified in 40 cycles (denaturation 95 °C 15 s, annealing 57 °C 30 s, extension 72 °C 30 s). A melting curve confirmed the amplification of a single product. Relative gene expression was calculated using two reference genes [PPIA and RPLP0 (ARBP)] according to the formula:

$$Relative\, gene \,expression= \frac{{({E}_{GOI})}^{\Delta CtG\, OI}}{GeoMean[{\left({E}_{REF}\right)}^{\Delta Ct\, REF}]}.$$

To test for significance, one-way ANOVA with post-hoc Bonferroni correction was conducted using GraphPad Prism Software (v8.1.2). The null hypothesis was accepted for α < 0.05.

### Southern blot

To evaluate the integration of the reprogramming vector coMIP247 into the NWR genome, genomic DNA was extracted from NWR fibroblasts, and NWR iPSCs harvested at passage P2, P12 and P15 using the DNeasy Blood & Tissue Kit (Qiagen, #69506) following the manufacturer’s instructions. 10 µg of NWR genomic DNA and coMIP247 control DNA were digested with SPE1-HF (NEB, # R3133L) at 37 °C overnight. Digested DNA was subjected to gel electrophoresis at 55 V for 7 h on a 0.8% LE GP agarose gel (Biozyme, #850070) containing 8 µg/ml ethidium bromide together with 2 µl 1 Kb DNA Ladder (Invitrogen, #15615-016) and 2 µl MassRuler DNA Ladder (ThermoFisher, #SM0403). After image acquisition including an UV ruler, the gel was placed in 0.125 M HCl for 10 min to depurinate DNA. After washing twice in distilled water, the gel was incubated in denaturation solution (1.5 M NaCl, 0.5 M NaOH) for 30 min. For blotting of DNA, an Amersham Hybond XL membrane (VWR, # RPN203S) was shortly washed in water, subsequently incubated in denaturation buffer for 30 min, and then placed onto the gel. Capillary blotting with denaturation solution was preformed overnight. At the next day, wells were marked with pencil and the DNA was crosslinked to the membrane using a StrataLinker1800 Crosslinker (Stratagene) with 100 × 100 µJ.

The southern blot probe was generated using the Prime-It II Random Primer Labeling Kit (Agilent, #300385) with alpha-dCTP32 on a 689 bp PCR product (part of dTomato sequence) amplified with AmpliTaq-Gold 360 DNA Polymerase (ThermoFisher, #4398790) from the coMIP247 plasmid using primers SB_Probe_F (AGGAGGTCATCAAAGAGTTCAT) and SB_Probe_R (TTACTTGTACAGCTCGTCCAT). The probe was hybridized to the blotted DNA in Amerhsam Rapid-hyb buffer (Cytiva, #RPN1635) at 65 °C overnight. Afterwards the membrane was washed twice for one hour in washing solution (0.3 M NaCl, 40 mM Tris/HCl, 2 mM EDTA, 0.2% SDS). A phosphor screen (Cytiva, # BAS-IP MS 2040 E) was exposed to the blot overnight and imaged on a FLA7000 imager (FujiFilm).

### RNA sequencing

For RNA sequencing of NWR iPSCs, total RNA was isolated using RNeasy mini kit (Qiagen, #74104) according to the manufacturer’s instructions. 3 µg of isolated RNA was treated with TURBO DNase (Life Technologies, #AM2239) followed by clean up using RNeasy MinElute RNA cleanup kit (Qiagen, #74204). RNA purity and quality were assessed with microcapillary electrophoresis on Agilent 2100 Bioanalyzer (RNA Pico 6000 kit, Agilent, #5067-1513). Ribosomal RNA (rRNA) was depleted from 1 µg of total RNA using RiboZero Gold (Human/Mouse/Rat) kit (Illumina), followed by purification with RNeasy MinElute RNA cleanup kit. Sequencing libraries were prepared from rRNA-depleted RNA using TruSeq Stranded total RNA LT kit (Illumina, #20020598) according to the manufacturer’s instructions. The final PCR amplification included 11 cycles followed by purification with Ampure XP beads (Beckman-Coulter, #A63881).

For RNA sequencing of SWR ESCs, total RNA was isolated using RNeasy micro kit (Qiagen, #74004) and subjected to library construction using NEBNext Ultra II Directional RNA Library Prep Kit for Illumina (NEB, #E7760L) according to the manufactuer’s instructions. cDNA was enriched by PCR in 12-cycles.

Quality of final libraries was evaluated by Agilent 2100 Bioanalyzer using DNA 1000 (Agilent, #5067-1504) or High Sensitivity DNA (Agilent, #5067-4626) kits. DNA concentrations were measured with Qubit (Life Technologies) and dsDNA HS Assay Kit (ThermoFisher, #Q32854). Libraries were sequenced using Illumina NextSeq 500 instrument [75-nt single-end reads, NextSeq 500/550 High Output Kit v2.5 (75 Cycles), Illumina, #20024906].

### Gene annotation of NWR transcriptome

Due to the unavailability of an assembled NWR genome, we retrieved the first version of the SWR genome (Ceratotherium Simum, cerSim1) from NCBI^56^. The genome has a total length of 2464.37 Mb and it was assembled to a total of 3087 scaffolds with a N50 of 26.27 Mb.

The last SWR transcriptome version was generated in June 2013 and contains 16,583 genes annotated by aligned human CDS from Ensembl v.72 and identified SWR proteins in Uniprot^57^. Hence, we annotated a new transcriptome by retrieving the total set of protein-coding and non-coding aligned human, mouse, and horse RefSeq genes from UCSC^58^. SWR genes were named using the corresponding human gene ID if the RefSeq homolog was found; otherwise they were named using the corresponding mouse or horse gene ID. We finally complemented the annotations by including the remaining SWR genes from the aforementioned annotated transcriptome, provided that human, mouse, and horse Refseq homologs were not found. Overall, we generated a new transcriptome composed of 28,789 genes. However, given the strong fragmentation of the SWR genome (> 900,000 scaffolds as compared to < 500 scaffolds in human), up to 25% of the annotated genes in the NWR transcriptome shared the same ID across two or more loci from different scaffolds, indicating partially assembled genes that were found in more than one contig or high sequence identity of genes or pseudogenized paralogs. For these cases, we generated unique gene IDs by attaching the name of the scaffold and the strand to the previous gene ID.

### Read mapping

RNA sequencing reads were clipped for residual adapter sequences and trimmed for low-quality 3′ ends. For mapping, we used STAR (v.2.7.1a)^59^ to align all RNA-sequencing data to the SWR genome. Maximum number of mismatches was set up to 4 to account for subspecies variability. Multiple mapping to several locations was allowed unless otherwise stated. We quantified the gene expression by using HTSeq^60^ and we filtered out genes with an average FPKM (fragments per kilobase per million mapped reads) lower than 1. Read counts were normalized by sample by applying DESeq2’s median of ratios^61^.

### Comparison of NWR transcriptome profiles against human, mouse and SWR ESCs

To compare the gene expression signatures of NWR iPSCs and SWR ESC with human and mouse PSCs, we downloaded additional available RNA-sequencing datasets for several human and mouse ESCs cultured in naïve and primed conditions (see Supplementary Table T3 for details). Processing, mapping to hg38 and mm10 genomes, and gene quantification to Ensembl v. 85 (human) and 87 (mouse) annotation releases were performed using the same methods as previously described for the NWR and SWR datasets. Next, we generated principal component analyses (PCAs) of the gene expression across species by selecting orthologs with a similar annotated gene name, and consistent expression in all analyzed species. In order to correct for differences in gene length across species, PCAs were displayed by using logFPKM counts, as these values are normalized by both gene length and library depth.

### Statistical RNA-sequencing data analyses and plots

Analysis of RNA-sequencing data, including generation of plots and statistical test, was done using R. The sets of differentially expressed genes between naïve (N2B27 and RSeT) and primed conditions were calculated by running DESeq2 using a cut-off of |FC|> 1.5 and an adjusted P value < 0.01. Significant KEGG pathways in the set of differentially expressed genes (naïve N2B27 vs. primed) were identified by using the function ‘gost’ of the gprofiler2 package^62^ with a P value < 0.05. Gene Set Enrichment Analysis (GSEA) was performed by running GSEA v4.0.3^63,64^ with N2B27 specific (N2 intercept, Fig. 5a, 944 genes) vs. all remaining genes using a set of 119 genes corresponding to the GO term meiosis I (GO:0007127).

### Ethics approval

All procedures involving mice were approved by the local authority (LAGeSo, Berlin) and performed under the license G0200/12-43.

The human cell line BIHi005-A is registered in the European Human Pluripotent Stem Cell Registry (hPSCreg: http://hpscreg.eu/cell-line/BIHi005-A) and has the Ethical committee approval number: 350 (Panel: 3) Protocol ID: 17576 from Stanford university. The registered information provides evidence that: (A) The line has been derived with full informed consent of the donor, and (B) No undue inducement has been provided for donation.

The NWR skin biopsy was taken from the female NWR Nabire at the ZOO Dvůr Králové, Czech Republic by an authorized veterinary, and shipped with the according CITES documents to our laboratory for fibroblast isolation.

## Supplementary Information

Supplementary Information 1.

Supplementary Information 2.

Supplementary Movie 1.

## Data Availability

The NWR RNA-sequencing data generated during this study are available at GEO: GSE161173. There are restrictions to the availability of SWR RNA-sequencing data as they are part of another publication (Hayashi et al., in preparation).
