# Supplementary material for: Naïve-like pluripotency to pave the way for saving the northern white rhinoceros from extinction
Source: Sci Rep. 2022 Mar 8;12:3100. doi: 10.1038/s41598-022-07059-w (PMC8904600; doi:10.1038/s41598-022-07059-w)
Supplement: Supplementary file 1 — Supplementary Information 1. [file 41598_2022_7059_MOESM1_ESM.docx]

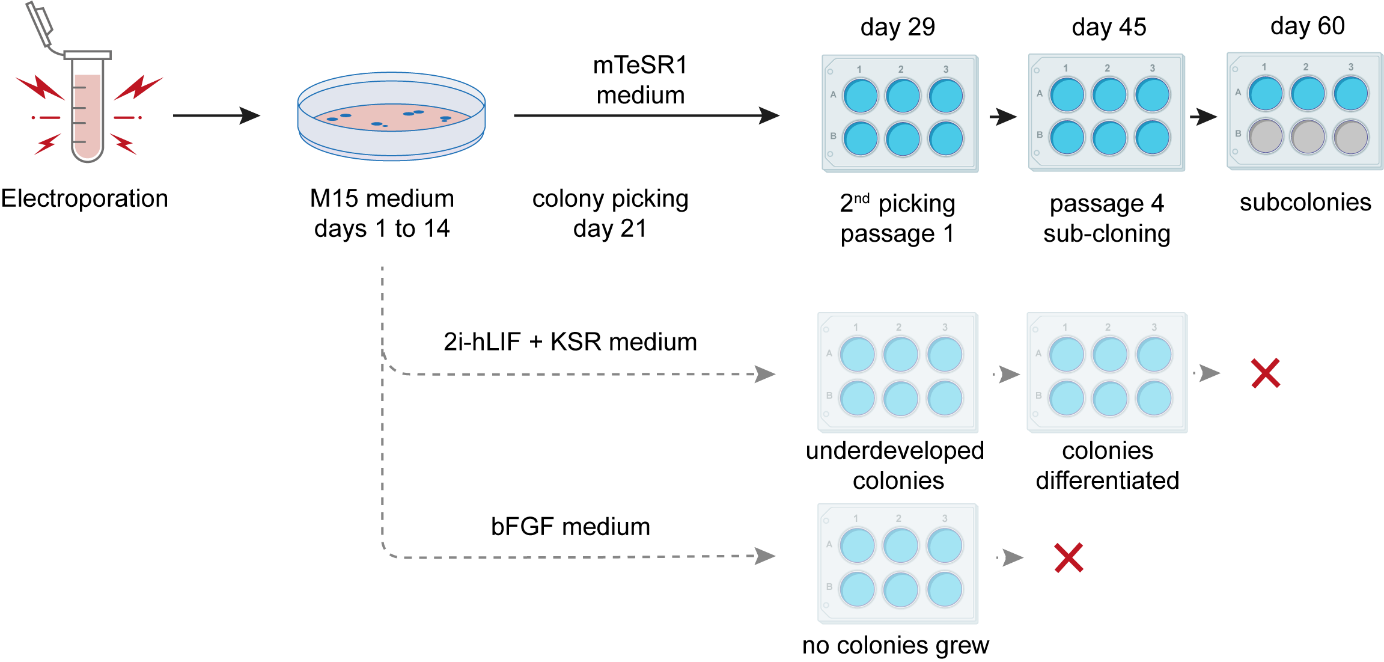


**Supplementary Figure S1.** **Overview of tested reprogramming conditions to generate NWR iPSCs.** Related to Figure 1.

After electroporation, cells were grown in M15 reprogramming medium containing LIF. After two weeks, the medium was changed to pluripotency supporting conditions, and at day 21 single colonies were picked. In total, three different media were tested: mTeSR1 (high bFGF medium) resulted in colony formation and successful line establishment as represented in Figure 1d; 2i-hLIF medium led to colony formation, but post picking no lines could be established (experiment terminated at day 45); (low) bFGF medium did not enable colony formation (experiment terminated at day 40).

**
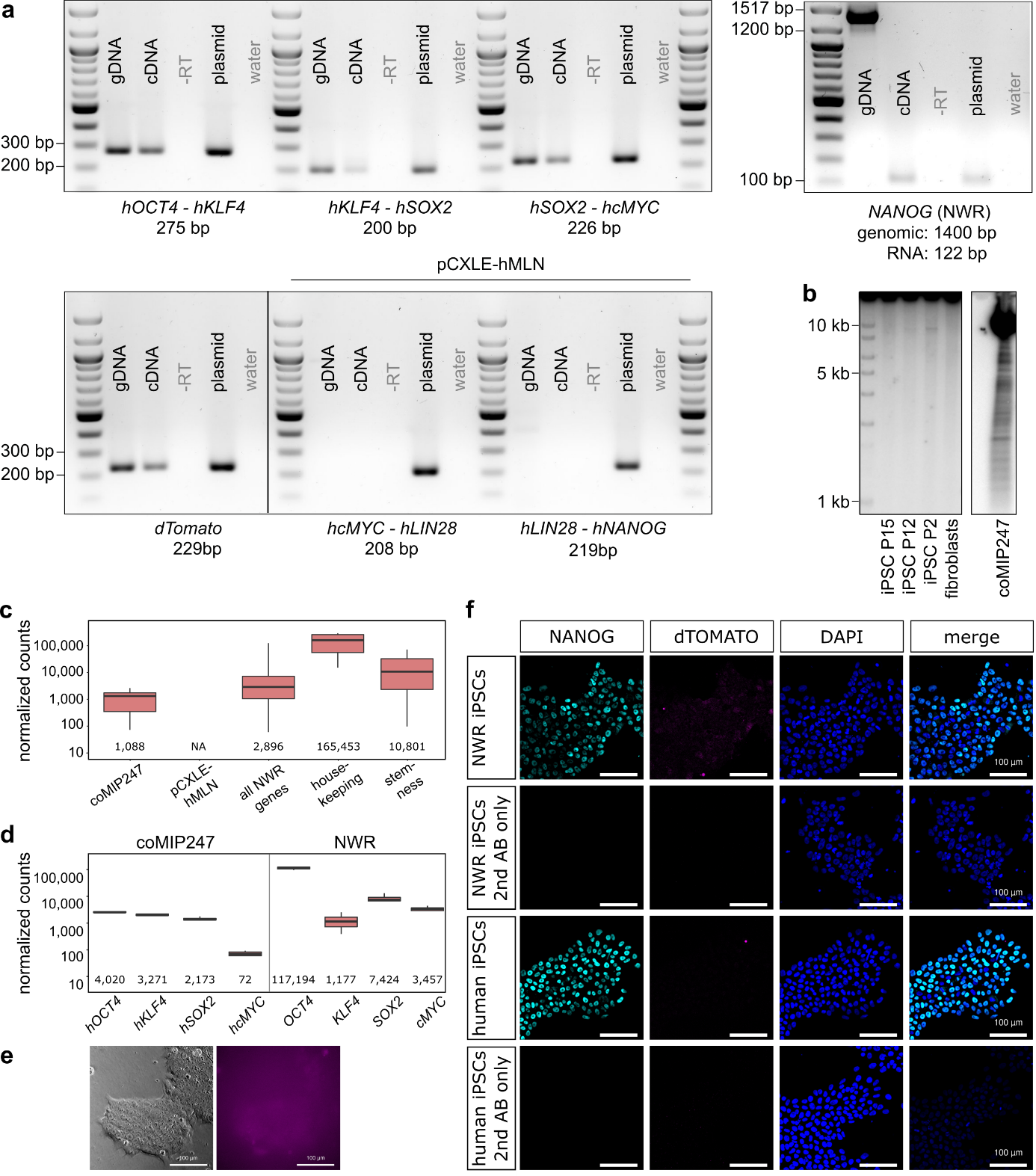
**

**Supplementary Figure S2.** **Integration and expression of the reprogramming vector coMIP247.** Related to Figure 1.

1. Gel-electrophoresis of PCR amplified genomic DNA (gDNA) and complementary DNA (cDNA). Primer spanned linker regions within the reprogramming vectors coMIP247 and pCXLE-hMLN to disable amplification of endogenous sequences. As negative controls, water and reverse transcription reaction lacking reverse transcriptase (-RT) were used. Plasmid DNA served as positive control. Amplification of endogenous NWR *NANOG* using exon spanning primers confirmed purity of gDNA and cDNA.
2. Southern blot of SPE1-HF digested genomic DNA isolated from NWR fibroblasts and iPSCs at passage P2, P12 and P15 revealed a single band in iPSCs but not fibroblasts indicating integration of coMIP247 at one side in the genome (left panel, exposure time: 60 hours). As control, digested coMIP247 DNA was loaded (right panel, exposure time: 15 minutes). Full-length blots are presented in Supplementary Figure S3.
3. In RNA-sequencing experiments, reads mapping to coMIP247, but not pCXLE-hMLN, were detected confirming the expression of coMIP247 in NWR iPSCs at approximately 1/3 and 1/10 of all expressed NWR and endogenous NWR genes associated with stemness, respectively. Numbers represent the median per gene category. Number of samples: four. Sample type: NWR iPSCs cultured in primed conditions (mTeSR1).
4. Expression of reprogramming factors encoded by coMIP247 in comparison to the expression of the corresponding endogenous NWR genes. RNA-sequencing data, numbers represent the median per gene. Number of samples: four. Sample type: NWR iPSCs cultured in primed conditions (mTeSR1).
5. Live imaging of NWR iPSCs. Left: phase contrast, right: dTOMATO expression, exposure time: 999ms. Scale bars: 100 µm.
6. NWR iPSCs stained for NANOG, dTOMATO, and secondary antibody only. As control, human iPSCs were stained in parallel. Scale bars: 100 µm.


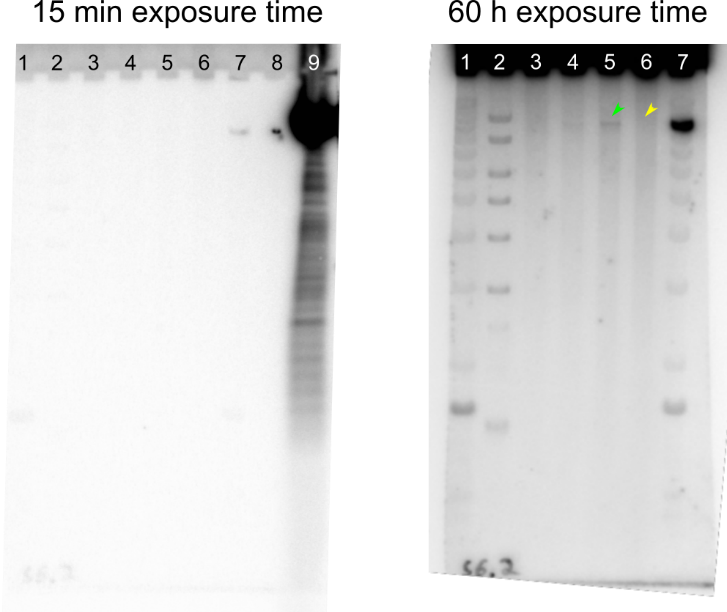


**Supplementary Figure S3. Full-length southern blot.** Related to Supplementary Figure S2b.

To test, if the reprogramming plasmid coMIP247 integrated into the NWR genome, we performed southern blotting of SPE1-HF digested DNA. Lanes were loaded as follows: Invitrogen 1 kb DNA Ladder (1), ThermoFisher MassRuler DNA Ladder (2), NWR iPSCs passage P15 (3), P12 (4), P2 (5), NWR fibroblasts (6), Invitrogen 1 kb DNA Ladder (7), empty lane (8), coMIP247 plasmid (9). coMIP247 gave a strong signal after 15 minutes exposure time (left). To enable longer exposure time, we cut the membrane between lane 7 and 8.

A single band in NWR iPSCs (green arrow), but not NWR fibroblasts (yellow arrow), became visible after 60 h exposure time (right). Signal in lane 7 (Invitrogen 1kb DNA Ladder) is probably an artefact from spilled coMIP247 plasmid (lane 9). As no signal was observed in lane 6 (NWR fibroblasts), we concluded that the signal in lane 5 (NWR iPSCs) is specific.


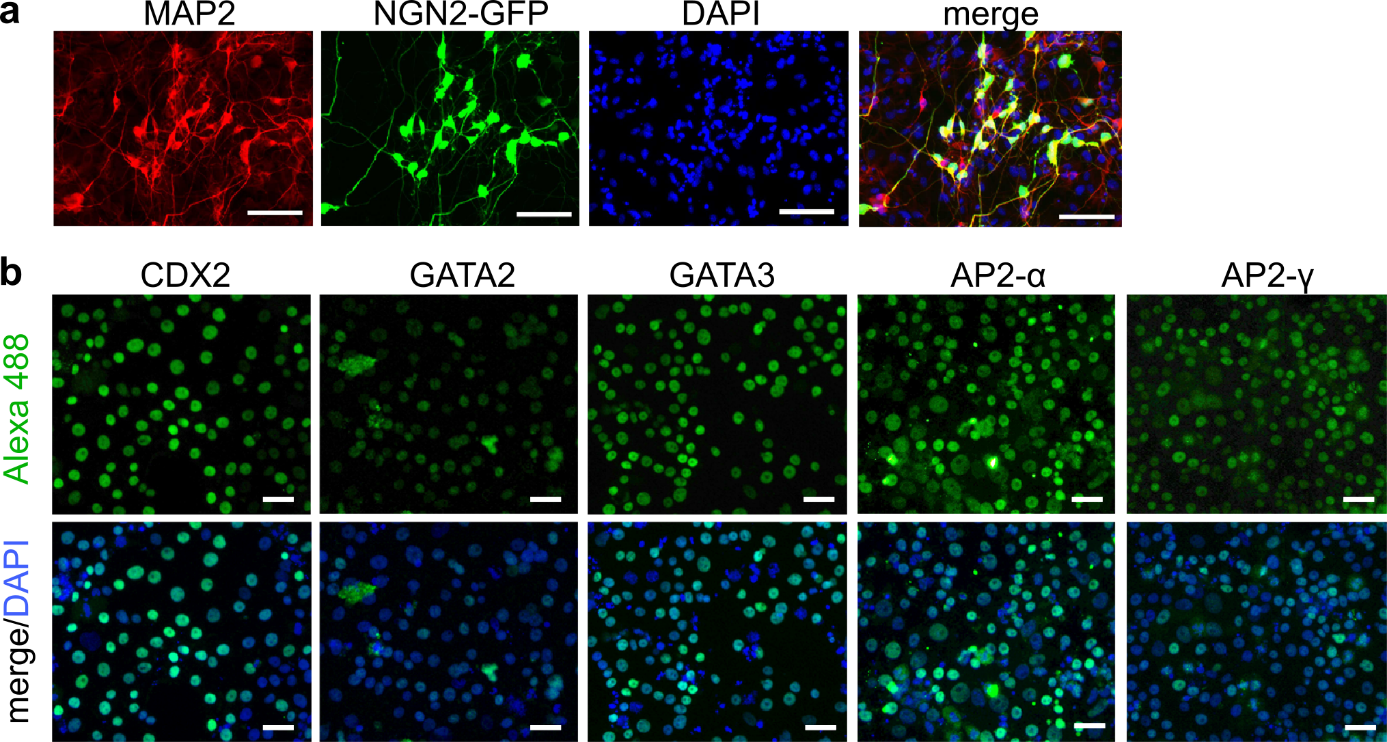


**Supplementary Figure S4. Immunostainings of NWR iPSCs** differentiated into forebrain-like neurons (using ectopic NGN2-GFP expression) **(a)**, and trophoblast progenitors **(b)**. Related to Figure 2. Scale bars: 50 µm **(a)** and 25 µm **(b)**.

**Supplementary Movie M1. NWR iPSCs differentiate into beating cardiomyocytes.** Related to Figure 2.

**
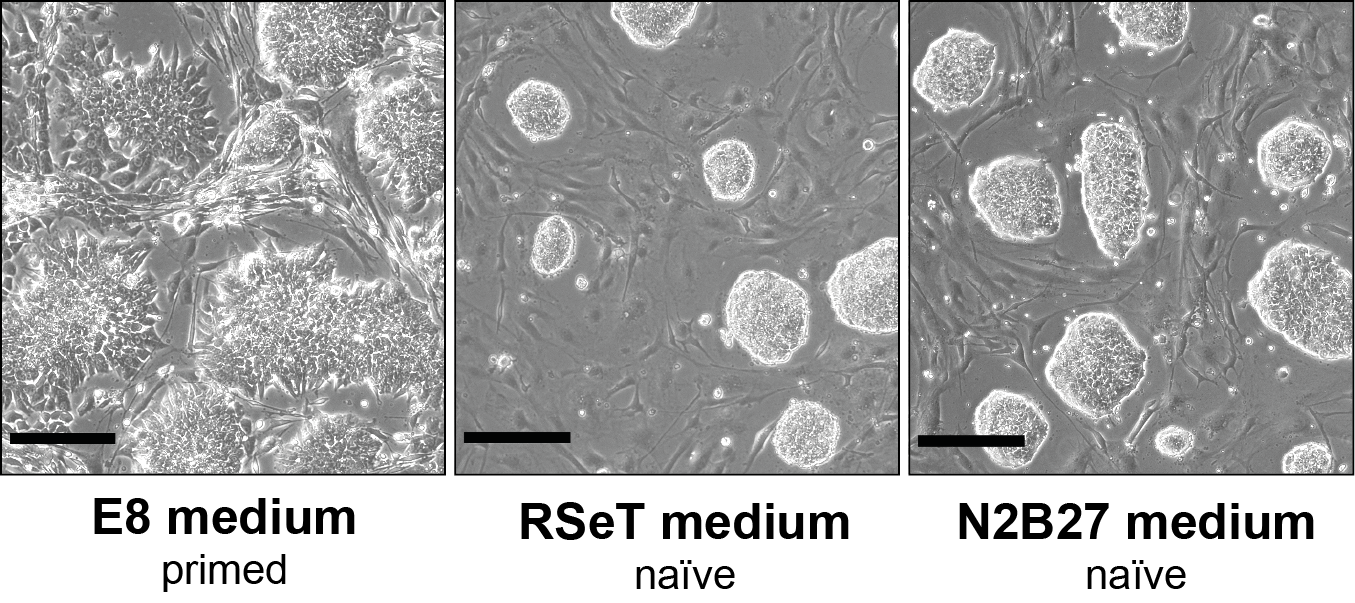
**

**Supplementary Figure S5. Morphology of human iPSCs in primed and naïve culturing conditions.** Related to Figure 3.

Human iPSCs (line BIHi005A) grown on mouse embryonic fibroblasts in primed (E8, left), and naïve conditions: RSeT (middle) and N2B27 (right). Scale bars: 100 µm.

**
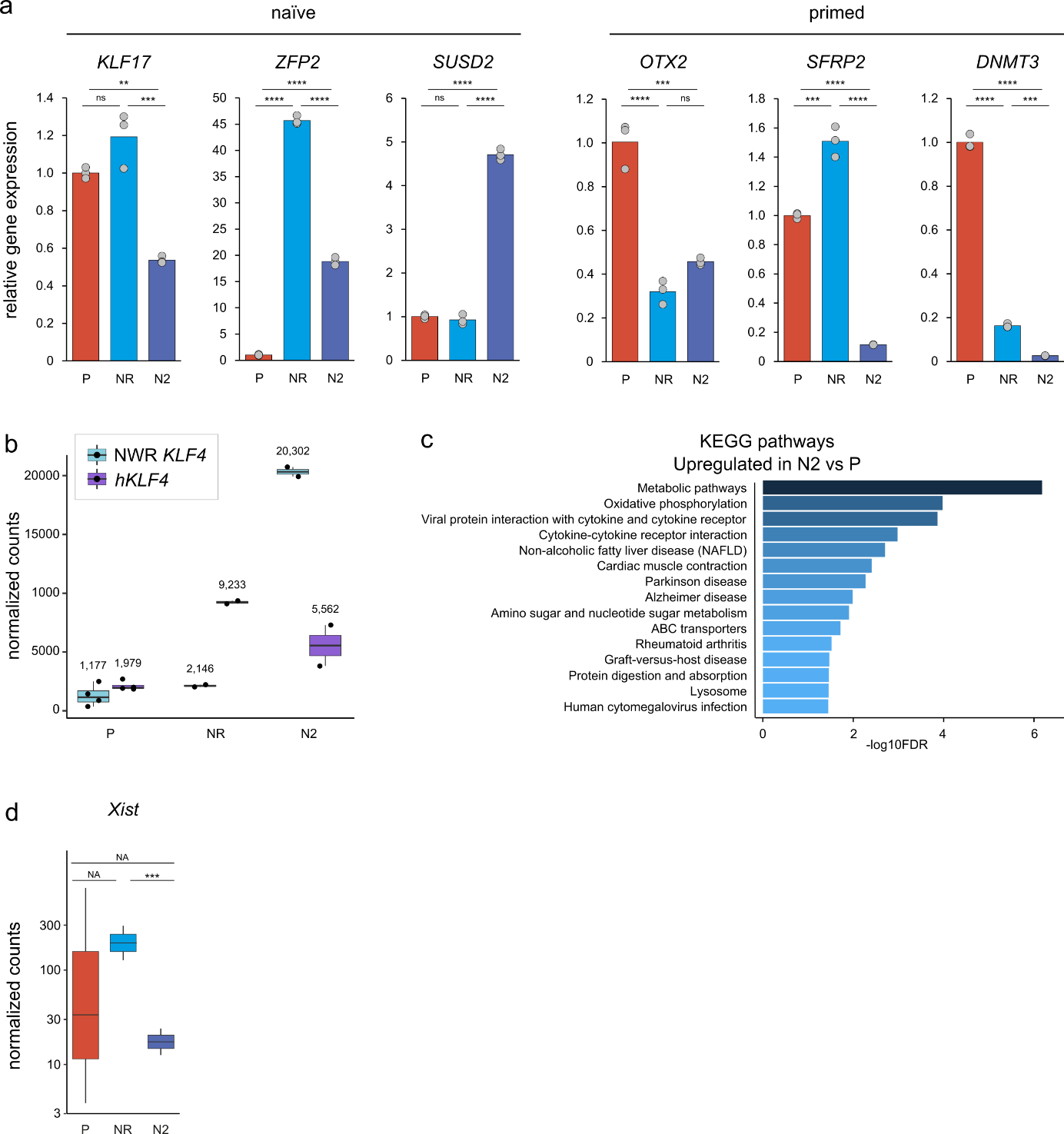
**

**Supplementary Figure S6. Characterization of naïve-like pluripotency in NWR iPSCs.** Related to Figures 3 and 5.

- 1. Relative expression of naïve and primed marker genes was measured by RT-qPCR. In 15/18 comparisons (P vs. NR, P vs. N2, NR vs. N2), results agreed with RNA-sequencing data (see Figure 3d). Bars represent means. *, **, ***, **** P value >0.05, 0.01, 0.001, 0.0001, respectively (one-way ANOVA followed by Bonferroni’s post-hoc test, α < 0.05).
  2. Expression of endogenous NWR *KLF4* and coMIP-encoded exogenous human *KLF4* (*hKLF*) in primed and naïve culturing conditions. RNA-sequencing data, numbers above bars represent the median.
  3. Differential gene expression analysis revealed 15 KEGG pathways, which were significantly (DESeq2 adjusted P value < 0.05) upregulated in naïve-like NWR iBCL2-GFP-iPSCs (N2, N2B27 protocol) as compared to primed NWR iPSCs (P, mTeSR1).
  4. The long non-coding RNA *Xist* is substantially and significantly lower expressed in N2 as compared to P and NR samples, respectively. The difference NR vs. N2 is significant according to DESeq2 (adjusted P value = 6.2 x 10^-10^). As *Xist* expression was not determined in one P sample and the variation was large in P samples in general, DESeq2 could not calculated P values for the comparisons P vs. NR and P vs. N2.

**Abbreviations**: ns: not significant; na: no answer; NR, N2: NWR iBCL2-GFP-iPSCs, naïve conditions, RSeT and N2B27 protocols, respectively; P: NWR iPSCs primed conditions (mTeSR1).

**Supplementary Table T1: List of key marker genes.** Related to Figures 3 and 5.

Marker genes associated with pluripotency, stemness, naïve and primed state, and for PGCs were selected from literature (i.a., ^1–4^). Additionally, seven common housekeeping genes were added to the list. Listed genes (60) were consistently detected in all NWR samples and used for comparison of gene categories across culturing conditions (Figure 3c). For gene expression comparison of NWR, SWR, human and mouse PSCs by principal component analysis only orthologous genes (excluding PGC marker genes), which were detected in all analyzed species, were used (46/60, black font, Figure 5d).

| **gene_name** | **gene_id** | **category** |
| --- | --- | --- |
| *ACTB* | *ACTB* | housekeeping |
| *CTNNB1* | *CTNNB1* | housekeeping |
| *EEF1A1* | *EEF1A1* | housekeeping |
| *GAPDH/JH767797.1/-* | *GAPDH* | housekeeping |
| *PPIA/JH767794.1/+* | *PPIA* | housekeeping |
| *RAF1* | *RAF1* | housekeeping |
| *RPLP0/JH767752.1/+* | *RPLP0* | housekeeping |
| *CFC1* | *CRIPTO* | stemness |
| *DNMT3A* | *DNMT3A* | stemness |
| *FGF4* | *FGF4* | stemness |
| *FGF5* | *FGF5* | stemness |
| *FZD5* | *FZD5* | stemness |
| *GRB7* | *GRB7* | stemness |
| *JARID2* | *JARID2* | stemness |
| *LIN28A* | *LIN28A* | stemness |
| *LIN28B* | *LIN28B* | stemness |
| *NODAL* | *NODAL* | stemness |
| *NOG* | *NOG* | stemness |
| *PTEN* | *PTEN* | stemness |
| *REST/JH767724.1/+* | *REST* | stemness |
| *SALL4* | *SALL4* | stemness |
| *TCF3* | *TCF3* | stemness |
| *TDGF1* | *TDGF1* | stemness |
| *ZFP42* | *ZFP42* | naïve |
| *DNMT3L* | *DNMT3L* | naïve |
| *DPPA5* | *DPPA5* | naïve |
| *ESRRB* | *ESRRB* | naïve |
| *IL6ST* | *IL6ST* | naïve |
| *KLF17* | *KLF17* | naïve |
| *KLF2* | *KLF2* | naïve |
| *KLF4* | *KLF4* | naïve |
| *KLF5* | *KLF5* | naïve |
| *TFCP2L1* | *TFCP2L1* | naïve |
| *SUSD2* | *SUSD2* | naïve |
| *BEX1* | *BEX1* | primed |
| *CD24* | *CD24* | primed |
| *DNMT3B* | *DNMT3B* | primed |
| *OCT6* | *POU3F1* | primed |
| *OTX2/JH767740.1/-* | *OTX2* | primed |
| *SFRP2* | *SFRP2* | primed |
| *TEAD2* | *TEAD2* | primed |
| *ZIC2/JH767732.1/+* | *ZIC2* | primed |
| *CD9* | *CD9* | pluripotency |
| *FOXD3* | *FOXD3* | pluripotency |
| *FUT4* | *FUT4* | pluripotency |
| *GABRB3* | *GABRB3* | pluripotency |
| *LEFTY1* | *LEFTY1* | pluripotency |
| *LIFR* | *LIFR* | pluripotency |
| *MYC* | *MYC* | pluripotency |
| *NANOG* | *NANOG* | pluripotency |
| *PODXL/JH767754.1/+* | *PODXL* | pluripotency |
| *POU5F1* | *POU5F1* | pluripotency |
| *SOX2* | *SOX2* | pluripotency |
| *TERF1* | *TERF1* | pluripotency |
| *TERT* | *TERT* | pluripotency |
| *TFAP2C* | *TFAP2C* | PGCs |
| *BLIMP1* | *BLIMP1* | PGCs |
| *PRDM14* | *PRDM14* | PGCs |
| *NANOS3* | *NANOS3* | PGCs |
| *STELLA* | *STELLA* | PGCs |

**Supplementary Table T2. Overview of blastocyst injections followed by retransfer of embryos into pseudo-pregnant foster mice.** Related to Figure 4.

Depicted are the number of retransferred embryos, which were either injected with NWR iPSCs or non-injected (control). Pseudo-pregnant foster mice were sacrificed at the indicated developmental stages (E7.5, E8.5 or E9.5). The number of deciduae reflects how many of the retransferred blastocysts implanted, and gives information about the success of retransfer. The number of isolated embryos shows how many embryos developed and were analyzed for GFP expressing cells. Embryos containing GFP positive cells were considered NWR-mouse chimeras. In one experiment (NWRN-iGFP-iPSCs, primed, E7.5), two embryos were isolated from one decidua.

|  | **Embryonic Day** | **Retransferred Embryos** | **Deciduae** | **Isolated Embryos** | **Chimeras** | **Success of retransfer**  **(% Deciduae/**  **Transferred Embryos)** | **Development**  **(% Isolated Embryos/ Deciduae)** | **Chimerism**  **(% Chimeras/**  **Isolated Embryos)** |
| --- | --- | --- | --- | --- | --- | --- | --- | --- |
| **Control**  (non-injected) | 7,5 | 23 | 17 | 12 | - | 73,9 | 70,6 | 0,0 |
|  | 8,5 | 25 | 20 | 17 | - |  | 85,0 | 0,0 |
|  | 9,5 | 48 | 41 | 26 | - | 87,5 | 61,9 | 0,0 |
| **NWR iGFP-iPSCs**  primed mTeSR1  P1 | 7,5 | 12 | 9 | 10 | - | 75,0 | 111,1 | 0,0 |
|  | 8,5 | 12 | 12 | 9 | 1 | 100,0 | 75,0 | 11,1 |
|  | 9,5 | 24 | 20 | 12 | 1 | 83,3 | 60,0 | 8,3 |
| **NWR iBCL2-GFP-iPSCs**  primed mTeSR1  P2 | 7,5 | 12 | 9 | 6 | 1 | 75,0 | 66,7 | 16,7 |
|  | 8,5 | 12 | 12 | 11 | - | 100,0 | 91,7 | 0,0 |
|  | 9,5 | 24 | 19 | 13 | 1 | 79,2 | 68,4 | 7,7 |
| **NWR iBCL2-GFP-iPSCs**  naïve N2B27  N2 | 7,5 | 12 | 8 | 4 | 2 | 66,7 | 50,0 | 50,0 |
|  | 8,5 | 12 | 11 | 4 | 4 | 91,7 | 36,4 | 100,0 |
|  | 9,5 | 24 | 21 | 6 | 1 | 87,5 | 28,6 | 16,7 |
| **NWR iBCL2-GFP-iPSCs**  naïve RSeT  NR | 7,5 | 12 | 9 | 4 | - | 83,3 | 40,0 | 0,0 |
|  | 8,5 | 12 | 10 | 5 | 1 | 91,7 | 45,5 | 20,0 |
|  | 9,5 | 12 | 8 | 2 | - | 66,7 | 25,0 | 0,0 |

**Supplementary Table T3. Published datasets of human and mouse ESCs.**

Overview of RNA-sequencing datasets obtained from human and mouse ESCs, cultured in naïve and primed conditions ^5–10^.

**Supplementary Table T4. Antibodies used for Immunofluorescence.**

| **Antibody** | **Marker for** | **Catalog #** | **Company** | **Dilution** |
| --- | --- | --- | --- | --- |
| rabbit isotype IgG | Control | GTX35035 | GeneTex | 1:200 |
| mouse isotype IgG | Control | 16-4714-85 | eBioscience | 1:200 |
| TFAP2A [AP-α] | Trophoblast | sc-184X | santa cruz | 1:100 |
| TFAP2C [AP-γ] | Trophoblast | sc-12762X | santa cruz | 1:100 |
| GATA2 | Trophoblast | sc-9008X | santa cruz | 1:100 |
| GATA3 | Trophoblast | sc-268 | santa cruz | 1:200 |
| CDX2 | Trophoblast | D11D10 | Cell Signaling | 1:40 |
| SOX2 | Pluripotency | CS1002 | Millipore | 1:100 |
| OCT4 | Pluripotency | 2840 (C30A3) | Cell Signaling | 1:100 |
| NANOG | Pluripotency | PA1-097  AF1997 | Thermo Fisher  R&D Systems | 1:100-1:200  1:100 |
| SSEA3 | Pluripotency | MA1-020 | Thermo Fisher | 1:100 |
| ACTN2 (α-Actinin) | Cardiomyocytes | A7811 | Sigma-Aldrich | 1:600 |
| TNNT2 (Troponin T) | Cardiomyocytes | A25973 (Human Cardiomyocyte Immunocyt-ochemistry Kit) | ThermoFisher | 1:1000 |
| GATA4 | Endoderm | D3A3M | Cell Signaling | 1:100 |
| GATA6 | Endoderm | D61E4 | Cell Signaling | 1:100 |
| SOX17 | Endoderm | 09-038 | Millipore | 1:100 |
| PAX6 | Neural stem cells | A24354  (Human Neural Stem Cell Immunocyto-chemistry Kit) | Thermo Fisher | 1:50 |
| NESTIN | Neural stem cells |  |  | 1:50 |
| SOX1 | Neural stem cells |  |  | 1:50 |
| SOX2 | Neural stem cells |  |  | 1:50 |
| MAP2 | iNeurons | 188004 | Synaptic Systems GmbH | 1:500 |
| dTOMATO/RFP | Transgene | biorbyt | orb334992 | 1:1000 |
| Alexa Fluor(R) 594 donkey anti-rabbit IgG (H+L) | 2^nd^ Antibody | A25970 | ThermoFisher | 1:200 |
| Alexa Fluor(R) 488 donkey anti-mouse IgG (H+L) | 2^nd^ Antibody | A25972 | ThermoFisher | 1:200 |
| Alexa Fluor(R) 488 goat anti-mouse IgG (H+L) | 2^nd^ Antibody | A11001 | ThermoFisher | 1:1000 |
| Alexa Fluor(R) 488 goat Anti-Rabbit IgG (H+L) | 2^nd^ Antibody | A11008 | ThermoFisher | 1:1000 |

**Supplementary Table T5. Primers.**

| **Primer name** | **Target** | **Sequence (5’-3’)** | **Product size (bp)** |
| --- | --- | --- | --- |
| hOCT-KLF4_F | coMIP247 Plasmid | ACTTCACCGCCCTGTACAG | 275 |
| hOCT4-KLF4_R |  | CTCCCGCCATCTGTTGTTAG |  |
| hKLF4-SOX2_F |  | GGCACTACAGAAAGCACACC | 200 |
| hKLF4-SOX2_R |  | CTTCAGCTCGGTTTCCATCA |  |
| hSOX2-cMYC_F |  | ATGAGCCAGCACTACCAGAG | 226 |
| hSOX2-cMYC_R |  | CCTCCTCGTCGCAGTAGAAA |  |
| dTOM_F |  | GCCCCGTAATGCAGAAGAAG | 229 |
| dTOM_R |  | GTGTAGTCCTCGTTGTGGGA |  |
| hcMYC-LIN28_F | pCXLE-hMLN Plasmid | GGAAACGACGAGAACAGTTGA | 208 |
| hcMYC-LIN28_R |  | GCCTCTTCTGCCGCCTTG |  |
| hLIN28-NANOG_F |  | CCTAGTGCACAGGGAAAGCC | 219 |
| hLIN28-NANOG_R |  | ACAAGCTGGATCCACACTCA |  |
| NANOG_ES_F | NWR NANOG | TCCAGCAGATGCAAGAACTTT | 122 (cDNA)  1400 (gDNA) |
| NANOG_ES_R |  | GCAAGTCTTTGGCCAGTTGT |  |
| PPIA_F | NWR PPIA | GTCCAGGAATGGCAAGACCA | 142 |
| PPIA_R |  | TCAAGCAGACGGGGGTAAAG |  |
| ARBP_F | NWR ARBP | CATGCTGAACATCTCGCCCT | 113 |
| ARBP_R |  | AGCGAGAATGCAGAGCTTCC |  |
| OTX2_F | NWR OTX2 | TGGGCTGGACATTCCAGTTT | 102 |
| OTX2_R |  | TCCTTCTATGCCTCCGGGAA |  |
| SFRP2_F | NWR SFRP2 | GTGGCTCAAAGACAGCTTGC | 91 |
| SFRP2_R |  | CTCCCCACCCACTTTCTGTC |  |
| DNMT3B_F | NWR DNMT3B | TGGAATACGAAGCCCCCAAG | 104 |
| DNMT3B_R |  | GACCAAGTACCCTGTCGCAA |  |
| KLF17_F | NWR KLF17 | TGTCTCCCTCCCAACCAAGA | 100 |
| KLF17_R |  | TTCCCATCAAAGGTCCTGGC |  |
| SUSD2_F | NWR SUSD2 | TTGGGACTTGTGCACTGTGT | 129 |
| SUSD2_R |  | CACTTTGTTGGGGTGCACAG |  |
| ZFP42_F | NWR SFP42 | CGAAGGGTGCGGAAAACGAT | 97 |
| ZFP42_R |  | GCCTTCAACGGGACACACAA |  |

**Supplementary References**

1. Habekost, M., Jørgensen, A. L., Qvist, P. & Denham, M. Transcriptomic profiling of porcine pluripotency identifies species-specific reprogramming requirements for culturing iPSCs. *Stem Cell Res.* **41,** 101645 (2019).

2. International Stem Cell Initiative *et al.* Characterization of human embryonic stem cell lines by the International Stem Cell Initiative. *Nat. Biotechnol.* **25,** 803–816 (2007).

3. Messmer, T. *et al.* Transcriptional Heterogeneity in Naive and Primed Human Pluripotent Stem Cells at Single-Cell Resolution. *Cell Rep.* **26,** 815–824.e4 (2019).

4. Hayashi, K., Ohta, H., Kurimoto, K., Aramaki, S. & Saitou, M. Reconstitution of the mouse germ cell specification pathway in culture by pluripotent stem cells. *Cell* **146,** 519–532 (2011).

5. Atlasi, Y. *et al.* The translational landscape of ground state pluripotency. *Nat. Commun.* **11,** 1617 (2020).

6. Chan, Y.-S. *et al.* Induction of a human pluripotent state with distinct regulatory circuitry that resembles preimplantation epiblast. *Cell Stem Cell* **13,** 663–675 (2013).

7. Dirks, R. A. M. *et al.* Allele-specific RNA-seq expression profiling of imprinted genes in mouse isogenic pluripotent states. *Epigenetics Chromatin* **12,** 14 (2019).

8. Factor, D. C. *et al.* Epigenomic comparison reveals activation of “seed” enhancers during transition from naive to primed pluripotency. *Cell Stem Cell* **14,** 854–863 (2014).

9. Rostovskaya, M., Stirparo, G. G. & Smith, A. Capacitation of human naïve pluripotent stem cells for multi-lineage differentiation. *Development* **146,** (2019).

10. Takashima, Y. *et al.* Resetting transcription factor control circuitry toward ground-state pluripotency in human. *Cell* **158,** 1254–1269 (2014).
